# Molecular Dynamics Simulations and Structural Analysis to Decipher Functional Impact of a Twenty Residue Insert in the Ternary Complex of *Mus musculus* TdT Isoform

**DOI:** 10.1371/journal.pone.0157286

**Published:** 2016-06-16

**Authors:** Eshita Mutt, Ramanathan Sowdhamini

**Affiliations:** National Centre for Biological Sciences (TIFR), GKVK Campus, Bellary Road, Bangalore 560 065, India; Centro de Biología Molecular Severo Ochoa (CSIC-UAM), SPAIN

## Abstract

Insertions/deletions are common evolutionary tools employed to alter the structural and functional repertoire of protein domains. An insert situated proximal to the active site or ligand binding site frequently impacts protein function; however, the effect of distal indels on protein activity and/or stability are often not studied. In this paper, we have investigated a distal insert, which influences the function and stability of a unique DNA polymerase, called terminal deoxynucleotidyl transferase (TdT). TdT (EC:2.7.7.31) is a monomeric 58 kDa protein belonging to family X of eukaryotic DNA polymerases and known for its role in V(D)J recombination as well as in non-homologous end-joining (NHEJ) pathways. Two murine isoforms of TdT, with a length difference of twenty residues and having different biochemical properties, have been studied. All-atom molecular dynamics simulations at different temperatures and interaction network analyses were performed on the short and long-length isoforms. We observed conformational changes in the regions distal to the insert position (thumb subdomain) in the longer isoform, which indirectly affects the activity and stability of the enzyme through a mediating loop (Loop1). A structural rationale could be provided to explain the reduced polymerization rate as well as increased thermosensitivity of the longer isoform caused by peripherally located length variations within a DNA polymerase. These observations increase our understanding of the roles of length variants in introducing functional diversity in protein families in general.

## Introduction

TdT is a unique eukaryotic DNA polymerase, which catalyzes *de novo* addition of random nucleotides to DNA primer in the presence of dNTPs and metal ions, thereby promoting antigenic variation in the vertebrate adaptive immune system (by V(D)J recombination [1–3] as well as non-homologous end-joining (NHEJ) pathways). This polymerase is a low-fidelity enzyme which does not require assistance from a complementary template strand. Overexpression of these TdT isoforms in cancerous (acute lymphoblastic leukemia/lymphoma, ALL) cells, emphasizing the importance of investigating the structural aspects of TdT and provide pointers for drug design [4–6].

**Fig 1:**
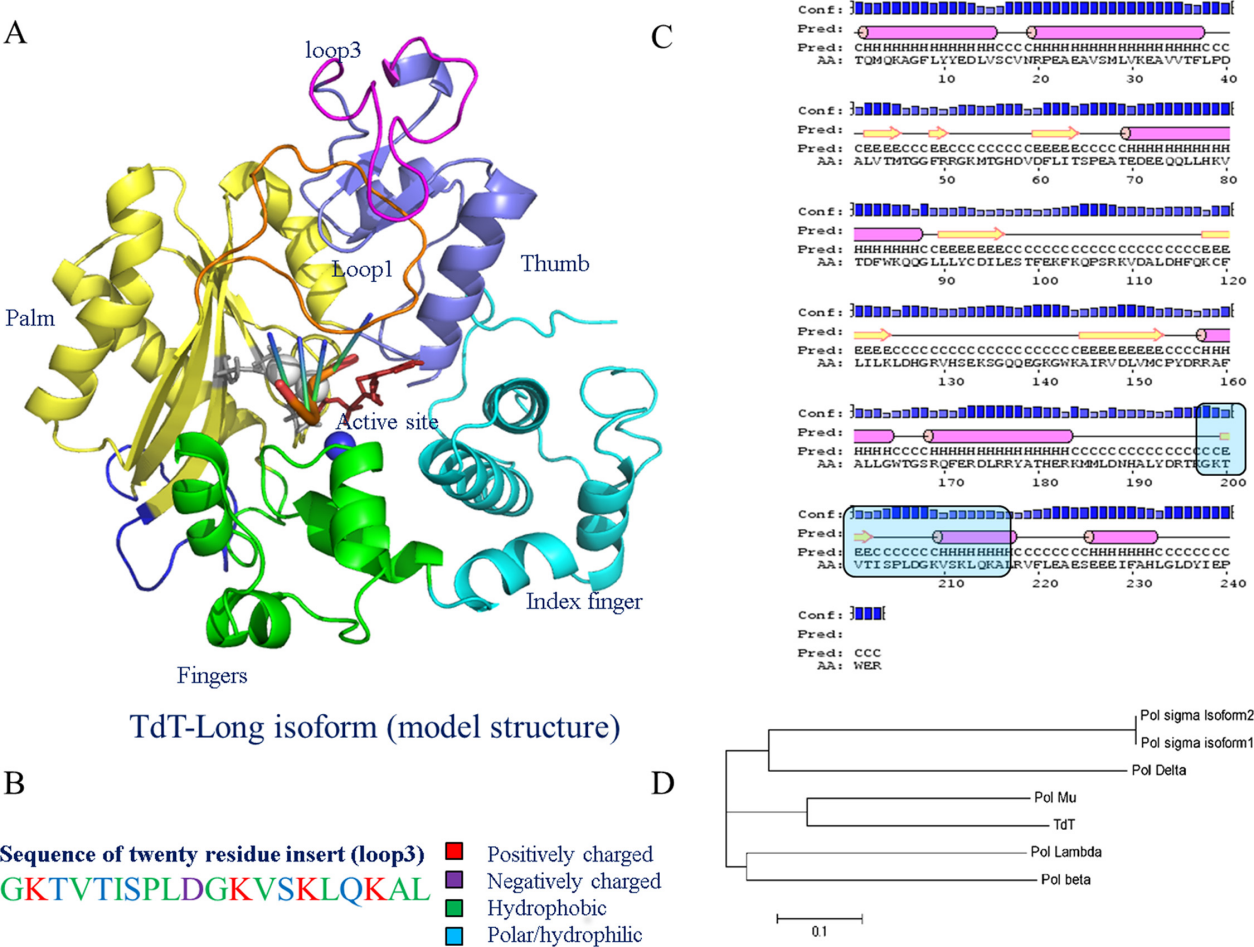
Structure and modeling of insert in TdT-long isoform. a) Doughnut shaped structure of TdT-long modeled isoform and its structural sub-domains. Colour index: Index finger (cyan), fingers (green), palm (yellow), thumb (blue), Loop1 (orange), Loop3 (magenta), Loop2 (blue) b) Sequence composition of Loop3. Color coded according to their physiochemical properties c) Secondary structure prediction of TdT-long form by PSIPRED, d) Phylogenetic clustering of TdT and other DNA polymerases in mouse showed that TdT and Pol mu shared a sequence identity of 42% and clustered together.

The three-dimensional structure of TdT resembles that of a typical polymerase fold, with the overall shape of a ‘hand’, where the N-terminal region (of 8kDa) forms the ‘index finger’ (residues 149–243), followed by helical ‘fingers’ subdomain (residues 243–302), the anti-parallel β-sheet containing ‘palm’ subdomain (residues 303–451) and finally the ‘thumb’ (452–510) subdomain at the C-terminal end (Fig 1A) [7]. Even though the secondary structural content of the catalytic domain (PDB code: 1JMS) is 46% helical, some loops are still functionally relevant (Fig 1A and 1B). While the palm region has the active site residues (Asp343, Asp345, and Asp434) and the lariat-shaped Loop1, the thumb region is responsible for correct placement and binding to DNA (similar to polymerase-μ and polymerase-λ, which depend on their thumb regions to secure DNA) [8]. Loop1 (position 382–401) is located over the active site and is mainly responsible for the template-independent property of TdT [4,5] (Fig 1A). Loop2 (412–428) is not involved directly in the catalysis, but is the most flexible part of TdT, as evident by the ill-defined portion of loop2 in the first reported crystal structure of TdT-short form (PDB code: 1JMS) [4].

Two isoforms of TdT have been identified in *Mus musculus*. A prominent, short isoform (510 amino acids; NCBI accession no: NP_001036693.1; TdT-short) and longer isoform (530 amino acids; NCBI accession no: NP_033371.2; TdT-long). The longer isoform has an insert of twenty residues (termed here as “Loop3” as per nomenclature in [6]) embedded in the “thumb” subdomain of TdT, thereby forming a projection over the Loop1 (as observed from our modelling efforts of Loop3) (Fig 1A–1C) [9]. Both the forms share all the features of a typical TdT scaffold that are essential for binding of incoming nucleotide, DNA primer, and metal ions. However, biochemical characterization of the two isoforms revealed reduced polymerization and increased thermosensitivity in TdT-long form compared to the smaller isoform, although they exhibit similar kinetic parameters [10,11] (Fig 1A and 1B). The longer isoform is stable at low temperatures, but is observed to be inactivated at even standard physiological temperatures (~37°C) [10]. Given the influence of neighbouring loops on the catalytic efficiency and substrate selection, we sought to examine the impact of length difference in loop regions of two isoforms of TdT. Inserts situated proximal to the active site or ligand binding site may directly inhibit the functionality of a protein, while distal inserts can also cause structural perturbations to regions far away from the insert position [12,13]. Thus, in order to evaluate the structural effects of the twenty-residue insert (“Loop3”), located distal from the active site on the TdT-long isoform, we have employed homology modeling and molecular dynamics simulation approaches.

## Methods

### Phylogenetic analysis of different DNA polymerases from mouse

In order to test the effect of the presence of inserts on the functionality of a protein, mRNA splice variants (protein isoforms) in different protein families from LenVarDB were screened [14]. Protein sequences of various mouse DNA polymerases were retrieved from NCBI and aligned using MUSCLE [15]. A phylogenetic neighbor-joining tree was constructed using MEGA [16]].

### Modeling of TdT-long form

The 20 residue insert (Loop3) is composed mostly of hydrophobic residues and has α-helical propensity within eight residues [Val495-Leu502] of the loop (Fig 1C). It also possesses positively charged residues which form a patch on the surface of thumb region in TdT (S1B Fig). The TdT ‘long’ isoform was modeled using TdT-short isoform as the template [PDB code: 1JMS]. Comparative modeling strategy for the 20 residue insert was not feasible due to lack of high sequence identity templates for that local region. The twenty residue insert (Loop3) was therefore introduced by PRODAT loop modeling module of SYBYL7.2 software package (Tripos Inc.) and iTASSER [17] (S1 Fig). Models with three best loop conformations of Loop3 were selected using different structural validation programs (HARMONY [18], VERIFY3D [19] and PROSA [20]). The best scoring model, where conformation of Loop3 was derived from iTASSER (referred henceforth as ‘iTASSER-model’) was selected as the starting structure for simulation studies of TdT-long form (S1C and S1D Fig). Different orientations of top-ranking Loop3, modeled by iTASSER and PRODAT library, have been illustrated in S1A Fig. Molecular dynamics studies were also performed for the top four ranking models of the long isoform (where alternate conformations of Loop3 are proposed, hereafter referred as “Loopmodel8”, “Loopmodel22” and Loopmodel23”). Binary complexes of TdT-short form (PDB code: 1KEJ, 1KDH) [6] were only available at the start of this study. Hence, the coordinates of DNA primer, ions and ddATP from the binary complexes were superposed onto TdT-short form (PDB code: 1JMS) to model the positions of these heteroatoms in the context of TdT-long isoform. Missing residues in Loop2 (GQQ motif) of both the forms were added using PyMOL [21]. The resultant complexes were further minimized by steepest descent till convergence in SYBYL7.2 software package (Tripos Inc.) and used as starting structures for molecular dynamics simulations and docking methods. Recently, ternary complexes of TdT-short form have been crystallized [22] and structural comparison with the superposed TdT-short form and modeled TdT-long form ternary complexes showed that the modeled structures are similar to the newly solved crystal structures.

### Molecular dynamics (MD) protocol

All-atom simulations with explicit solvent were performed at different timescales, varying from 30 ns to 100 ns, for a simulation time of 1910 ns (~2 μs) on the whole. All simulations were performed using GROMACS-4.5.5 program [23]. Five simulations and their replicates (to obtain statistical significance) were carried out for different temperatures: 298 K, 300 K, 311 K and 318 K for both TdT-short and TdT-long isoforms. Extended runs for the TdT longer isoform were also carried out for 300 ns (in duplicates). All simulations were carried out at neutral pH. Parameters for protein, DNA and nucleotide were utilized from the AMBER99SB-ILDN force field itself.

### Protocols for MD simulations

The starting protein structures were immersed into a cubic box with boundaries extending at least 10 Å in all directions from the protein periphery and filled with TIP4P water molecules to solvate the protein [24,25]. Appropriate number of counter ions was added to neutralize the total charge in the system. The system was minimized using steepest descent algorithm until it converged with *F*max no greater than 1000 kJ mol^−1^ nm^−1^. Equilibration of the protein was performed in two steps, firstly, for 500 ps under NVT and for 500 ps under NPT ensemble, while restraining the protein atomic positions. During equilibration, the coupling constant of the external bath was set to 0.1 ps for both protein and non-protein elements, where periodic boundary conditions were applied at constant temperature of 298 K (or 300 K, 311 K, 318 K appropriately), by using weak coupling with external bath (V-rescale method) [26] and constant pressure of 1 atm (Parrinello-Rahman pressure coupling) [27]. Bond lengths of heavy atoms were constrained using Linear Constraint Solver (LINCS) algorithm [28]. Coulomb and Van der Waals interactions were truncated at 10 Å and 14 Å, respectively, while long-range electrostatic interactions utilized Particle Mesh Ewald summation method [29]. Production runs ranging from 30 ns to 100 ns, with a timestep of 2 fs, were then performed upon the equilibrated systems using AMBER99SB-ILDN force field and the leap-frog algorithm. Snapshots of conformations were collected at every 500 ps. Production run at each temperature was replicated using different seed numbers for TdT-short and TdT-long isoforms. Parameters of substrate (ddATP) were obtained from PRODRG server [30].

### Mutation of Loop3

Loop3 (twenty residue insert) is positioned on the surface of TdT and possess charged residues, especially four lysines (Lys 484, Lys 494, Lys 496, Lys 500), which may be responsible for binding with Loop1 (Fig 1B and S1B Fig). These Lysine residues were mutated to Alanine and the above protocol for MD simulations was performed at 300 K for 100 ns on the mutated TdT-long isoform.

### Analysis of molecular dynamics results

Analysis of MD trajectories was performed using GROMACS tools. Secondary structural content and salt bridge calculations were performed by STRIDE [31] and salt bridge calculator in Timeline plugin of Visual Molecular Dynamics (VMD) package [32]. Overall deviation from the starting structure is measured by root mean square deviation (RMSD), and the resultant root mean square fluctuations (RMSF) were calculated by using the Cartesian coordinates of the C^α^ atoms. These values also highlight the regions which tend to fluctuate differentially with respect to other regions of the protein of interest. RMSD for TdT-short form stabilized within 5 ns, but it took around 20 ns for TdT-long form to stabilize. Hence, trajectories from 20 ns onwards have been considered for this analysis. Essential dynamics of simulation studies are useful for explaining independent and collective motions in proteins [33,34]. This method is applied here by calculating the co-variance matrix and deriving principal component analysis (PCA) of the trajectories, and further mapping the first two dominant modes onto TdT structure. PCA is often used to pinpoint conformational changes occurring at the global level and the initial modes (projection of simulation trajectory along largest eigenvector-eigenvalue pair) often represent maximum movements of the protein which could be functionally relevant. Correlation matrices derived from covariance matrices were calculated [35]. Distance between various entities of TdT structure, such as Loop1 and Loop3, is calculated by tracing distances over Glu382-Phe401 (Loop1) and Gly483-Leu502 (Loop3). Network properties of protein residues, namely highly connected nodes (hubs), group of connected residues (clusters) or rigid regions (cliques: all nodes in graph are directly connected to each other), have been shown to be helpful in implicating protein stability and tracing the path of inter-domain communications earlier [36,37] and also in analyzing unfolding processes by tracking the cluster sizes [38]. Protein structure residue network was created from the simulation trajectories, using each residue as node and non-covalent interactions within residues as edges, on the basis of C^α^-C^α^ distances of neighboring residues being less than 6.5 Å distance cutoff. Network analysis was performed by GraProStr [39] for final snapshots (100 ns) of both isoforms at 311 K. The dimensions of channel/pore formed by active site cavity was measured using PoreWalker [40].

## Results

Phylogenetic analysis of murine DNA polymerases showed that TdT co-clustered with polymerase μ (Fig 1D). Hence, structural and molecular dynamics studies, performed for the latter, has been used as guidance for this work [41–43]. TdT-long form has a two-fold lower polymerization rate and specific activity, in addition to increased thermosensitivity, compared to TdT-short form [10]. We employed molecular dynamics (MD) simulations at specified temperatures (please refer to methods for the details) to understand the role of Loop3 in mediating the functional differences between the two isoforms of TdT. These specific temperatures were derived from the enzyme activity assay and thermosensitivity assay performed elsewhere [10]. These temperatures, *viz*. 298 K, 300 K (room temperature), 311 K (1 degree higher than the observed inactivation temperature of TdT-long form) and 318 K (inactivation temperature of TdT-short form), were used during simulations along with ddATP, Mg^2+^ ions and DNA primer (4-mer stretch of cytosine bases) to replicate the environment created during the activity assay (Table 1). Apart from the iTASSER-model for TdT-long isoform, another three top-ranking models (Loopmodel8, Loopmodel22 and Loopmodel23, please see Methods for details) were subjected to MD simulations at 311 K for 100 ns to ensure that the results are not biased by the starting conformation of Loop3. RMSD analyses show that the Loop3 conformers converge structurally during the simulations [RMSD of all loopmodels can be viewed at https://figshare.com/s/bd555e824f36adc0ccbe]. Three out of four top-ranking Loop3 conformers with lowest energies and best validation scores were observed to have similar interactions as compared to the representative iTASSER model.

**Table 1:** List of simulation runs performed at different temperatures for TdT isoforms. Duration of simulation and root mean square deviation (RMSD) of simulations carried out. Extended runs of TdT-long form at 311 K for 300 ns yielded RMSD for run1: 3.2± 0.21 Å and run2 (duplicate): 3.86 ±0.35 Å. RMSD of replicate run is shown in brackets.

Simulation form	Temperature	Trajectory length (ns)	RMSD_100 ns (Å)
TdT-short form	298 K	100 ns	1.65
	300 K	100 ns	1.48
	311 K	100 ns(extended to 300 ns [replicate])	1.75 (1.99)
	318 K	100 ns	2.16
TdT-long form	298 K	100 ns	3.52
	300 K	100 ns	2.64
	311 K	100 ns (extended to 300 ns [replicate])	3.08 (3.69)
	318 K	100 ns	2.83
Mutated TdT-long[four Lys mutated to Ala in Loop3]	300 K	100 ns	3.89
TdT-long form(loop model 8)	311 K	100 ns	2.57 (2.35)

### Distal insert (Loop3) induces global conformational changes in TdT

**Fig 2:**
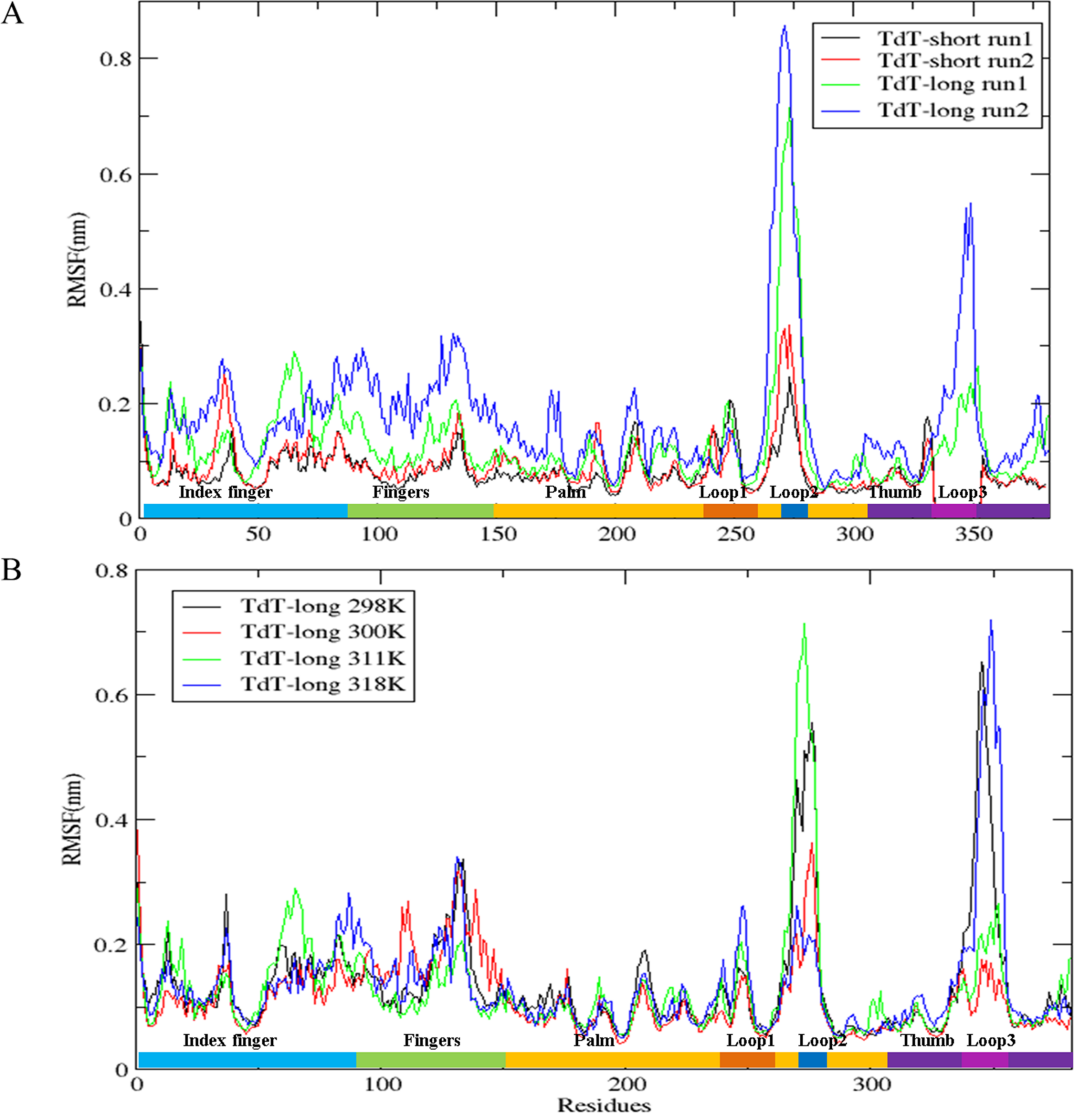
TdT-long form is more structurally dynamic than its shorter counterpart. a) Root Mean Square Fluctuations (RMSF) of TdT-short form and TdT-long form in its ligand bound state at 311 K. Index: TdT-short form (run1: black, run2: red), TdT-long form (run1: green, run2:blue). b) RMSF of TdT-long form at all simulation temperatures. Index: 298 K (black), 300 K (red), 311 K (green) and 318 K (blue). Sub-domains and loops of TdT has been marked on X-axis in different colours as: index finger (blue), fingers (green), palm (yellow), thumb(violet), Loop1 (orange), Loop2 (blue) and Loop3 (magenta)

**Fig 3:**
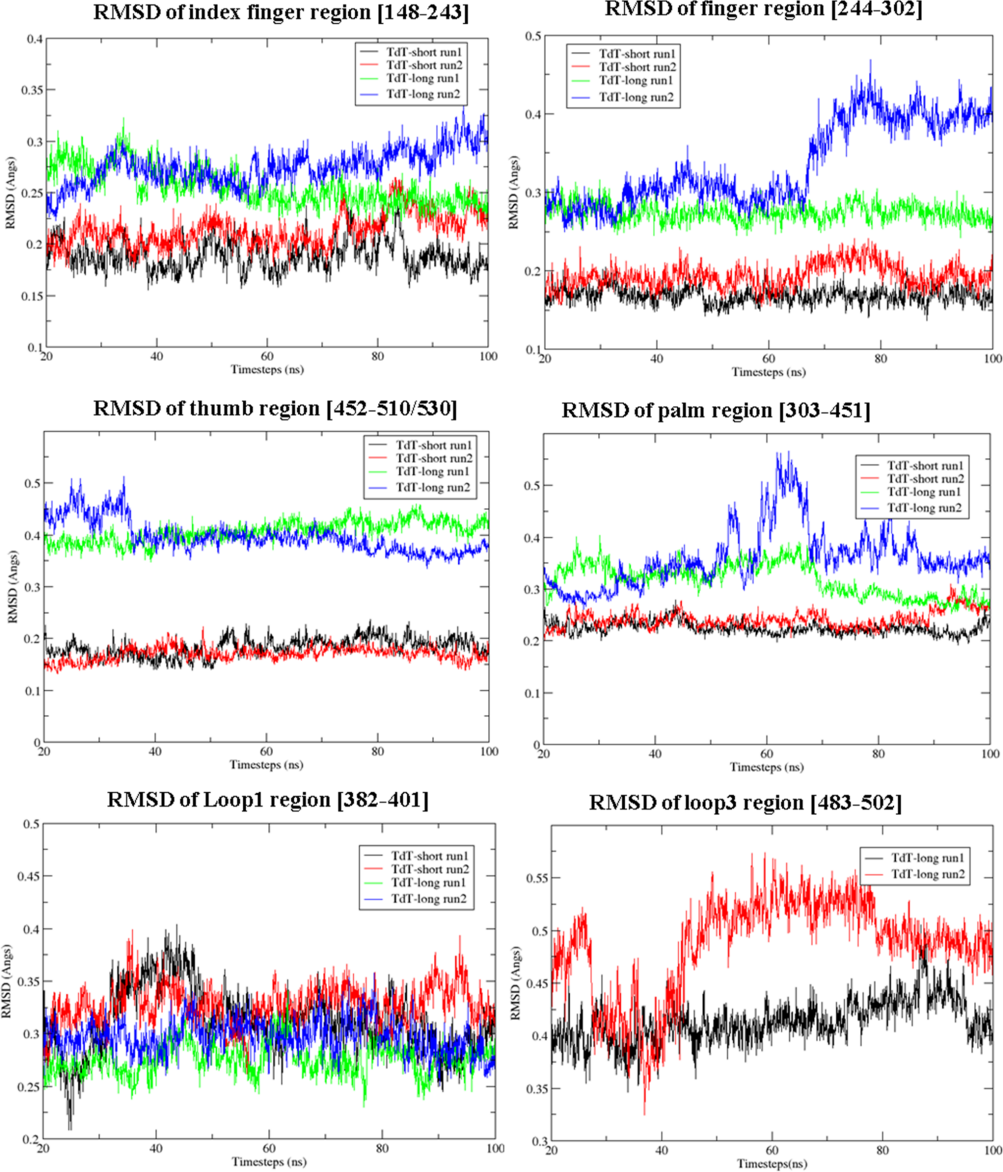
Deviations within different subdomains (except within Loop1) observed in TdT-short and long isoform. RMSD of subdomains of TdT-short and TdT-long for simulations runs at 311 K for 100 ns. Index: TdT-short (run1: black, run2: red), TdT-long form (run1: green, run2: blue).

RMSF calculations of TdT snapshots, derived from molecular dynamics simulations at physiological temperatures (300 K), showed that the ligand-bound state of TdT (protein bound with DNA primer, Mg^2+^ ions and ddATP) was more stable than the free enzyme state (S2A Fig). Subdomains like fingers and index-finger showed higher RMSF values in the TdT-long form, which may have been as a result of accommodation of the Loop3 insert (Fig 2A). At higher temperatures (nearer to inactivation temperature of TdT-short isoform), RMSF values for all subdomains in TdT-short isoform were slightly enhanced (S2B Fig). Higher temperatures (311 K, 318 K) entailed increased fluctuations at only Loop1, Loop2, fingers and index finger regions of TdT-long isoform. Loop3 was relatively more rigid at the physiological (300 K) and inactivation temperatures (311 K) (Table 2). Except Loop1, TdT long isoform showed higher structural deviations (calculated by RMSD values) compared to the shorter isoform (Fig 3, Table 3). The restricted movement of Loop3 (seen in both RMSD and RMSF) could be due to its spatial interaction with Loop1 at higher temperatures (Fig 2B). Lowered fluctuations of Loop1 in TdT-long form (both iTASSER-model and Loopmodel 8) imply that the presence of Loop3 is responsible for proximal as well as distal conformational changes in TdT-long isoform. However, the orientation of the modeled Loop3 (as seen in iTASSER-model and Loopmodel8 in S1A Fig) does not play a major role in these events. Loop2 was observed to possess maximum fluctuations among all subdomains but was not considered in the analysis due to its inherent thermal instability (as evident by the presence of high B-factor values in TdT crystal structure *viz*.1JMS.pdb).

**Table 2:** RMSF of each sector of hand-like structure of TdT *viz*. thumb, finger, index finger and loop1 (Increased RMSF values are marked in bold). Loop1 RMSF values reduce in TdT-long (wild and mutant). Simulations performed at 311 K for 100 ns except for the mutated TdT-long form.

Simulation form	RMSF-thumb	RMSF-finger	RMSF-index finger	RMSF-loop1
TdT-long run1	**1.30**	**1.23**	**1.56**	1.38
run2	**1.74**	**2.06**	**1.37**	1.21
TdT-short run1	0.95	1.00	1.17	**1.55**
run2	0.97	1.16	1.25	**1.31**
TdT-long mutated run1 (300 K)	**1.23**	**1.58**	**1.43**	1.16

**Table 3:** RMSD (Root Mean Square Deviation) from the starting equilibrated structure of each part of hand-like structure of TdT at 311 K, 100 ns.

Simulation form	Index finger	Finger	Palm	Thumb	Loop1
TdT-long run1	**2.55**	**2.74**	**3.17**	**4.06**	2.75
run2	**2.74**	**3.35**	**3.60**	**3.96**	2.96
TdT-short run1	1.88	1.68	2.24	1.82	**3.12**
run2	2.13	1.93	2.41	1.70	**3.26**
TdT-long mutated run1 (300 K)	**4.11**	**3.53**	**3.83**	**4.03**	2.86

### Structural rationale and possible role of Loop3 in increasing thermosensitivity of TdT-long isoform with respect to TdT-short isoform

According to our simulation studies, following series of events may explain the structural effects caused due to the Loop3 insertion and its resultant effect in elevation of thermosensitivity in TdT-long isoform.

#### Loop3 and Loop1 interact through electrostatic interactions

**Fig 4:**
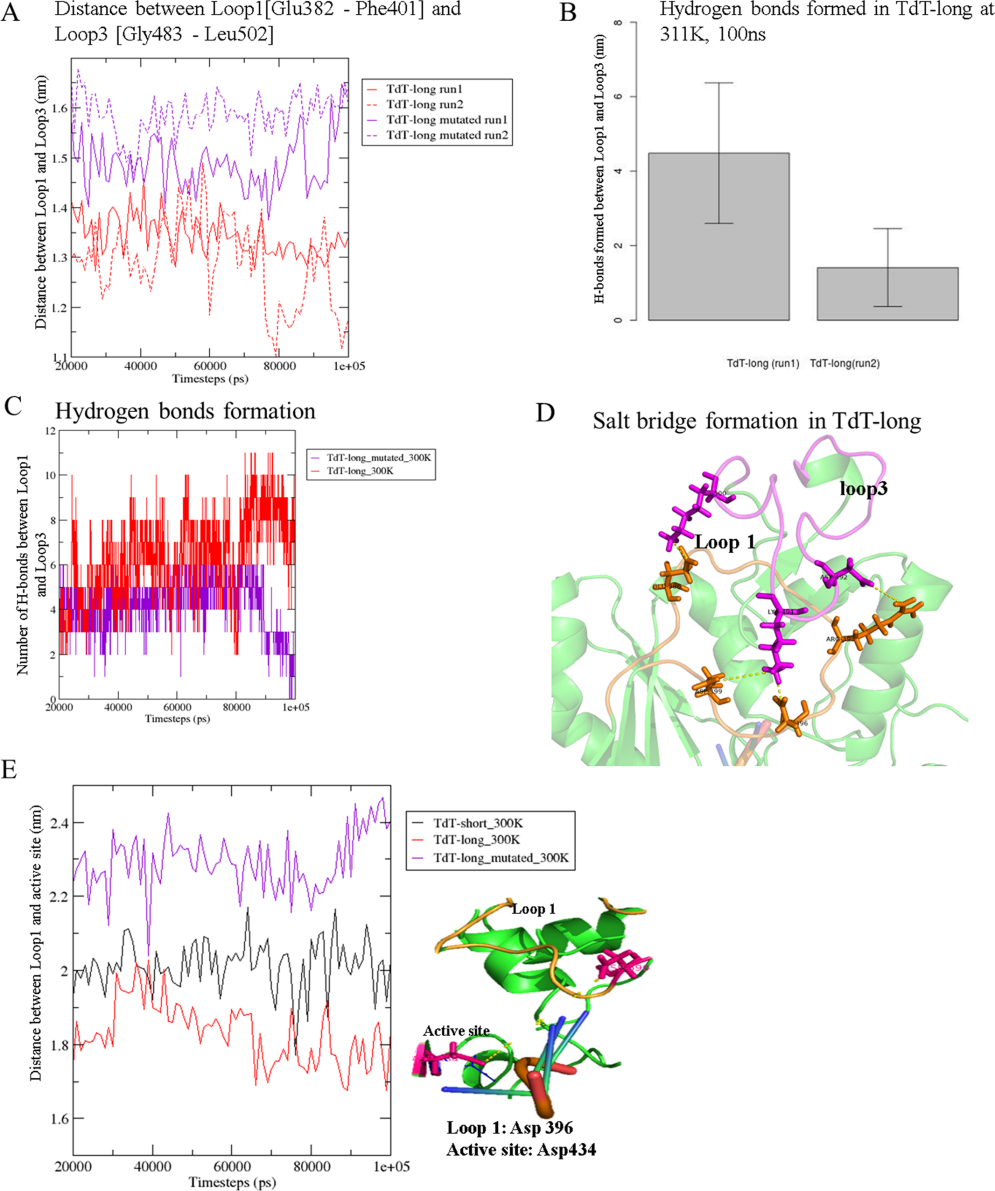
**Parameters to measure the interaction between Loop3 and Loop1** a) Distances between Loop3 & Loop1 of wild-type (red) and mutated (magenta) TdT-long form, b) & c) Number of hydrogen bonds formed during simulations of wild-type (red) and mutated (magenta) TdT-long form at 300 K, d) Salt bridges formed in wild-type TdT-long form but absent in mutated TdT-long form e) Left Panel: Distances between Loop1 (Asp396) and active site (Asp434) during simulations at 300 K in TdT-short form (black), wild-type TdT-long form (red) and mutated TdT-long form (magenta). Right Panel: Illustration of Loop1 projecting over active site.

**Fig 5:**
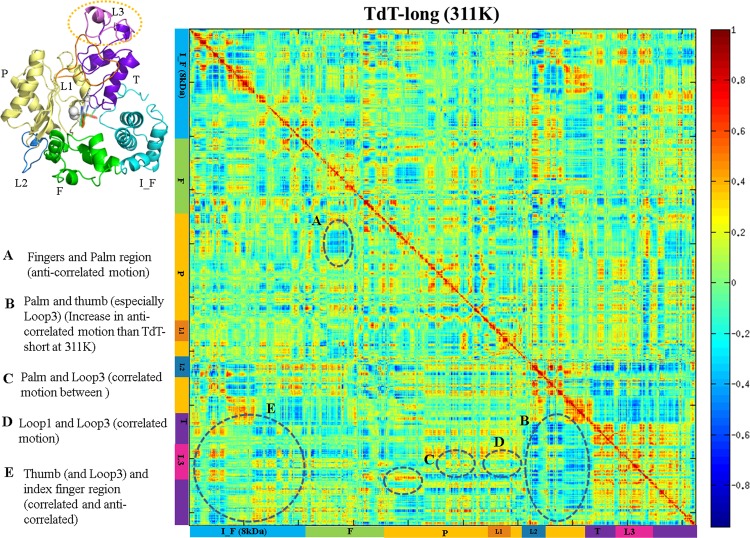
Inter-residue motions illustrated as cross-correlation matrices to depict rigid body motions within subdomains of TdT. Correlation matrix for TdT-long heavy atoms when simulated at 311 K. Positive values (red spectrum) indicate correlated motion; negative values (blue spectrum) indicate anti-correlated motions between sub-domains. Correlated and anti-correlated motions observed between sub-domains of TdT-long form have been marked by arrows. Sub-domains and loops of TdT has been marked on X-axis and Y-axis in different colours as: index finger or “I_F” (blue), fingers or “F” (green), palm or “P” (yellow), thumb or “T” (violet), Loop1 or “L1” (orange), Loop2 or “L2” (blue) and Loop3 or “L3” (magenta). Similar colour coded TdT-long structure has been provided at top left corner for ease in understanding.

Loop1 varies in length and function across DNA polymerases, but assumes a primary role in DNA template and substrate binding in Pol μ, Pol λ and TdT [44]. In the TdT-long form, Loop1 is spatially positioned in between the active site and Loop3 (Fig 1A). At higher temperatures, RMSF calculations showed that fluctuations in Loop3 reduced with increase in temperature (except at 318 K), while Loop1 fluctuated more at higher temperatures (311 K, 318 K) (Fig 2B). Spatial interactions, such as electrostatics (due to the presence of five charged residues in Loop3) and hydrogen bonds were observed between Loop3 and Loop1 during simulations (Fig 4A–4D) (Table 4). Mutation and further simulation of these charges led to the reduction of hydrogen bonds and absence of electrostatic interactions between Loop3 and Loop1 in Ala-mutant of TdT-long form (Fig 4A and 4C and S3 Fig). RMSF of Loop3 region in the mutated form was observed to be higher in comparison to wild type TdT-long form (RMSF wild-type TdT-long form 1.28 ±0.71 Å and mutated TdT-long form 1.57±0.82 Å). This shows that indeed charged residues and electrostatic interactions play an important role in the spatial organization of Loop3, as well as decreased fluctuations of Loop1 (S4A Fig). Distances traced across Loop1 and Loop3 were observed to be enhanced in the mutated TdT-long form (run1: 14.9±0.5 Å, run2: 15.8±0.4 Å), as compared to wild-type TdT-long form (run1: 13.4±0.4 Å, run2: 12.8±0.8 Å), implying that both loops may undergo a correlated movement (Figs 4A and 5 and S3 Fig). Further, conformational changes occurring in Loop1 with respect to the active site in presence/absence of Loop3 were also tracked (Fig 4E). Loop1 was observed to have restricted movement towards the active site in the TdT-long form, as compared to TdT-short form [please see details in S1 File].

**Table 4:** Percentage of occurrence of salt bridges between Loop3 and Loop1 throughout the simulations at 300 K and 311 K of TdT-long isoform.

Salt bridge pair	TdT-long [300 K]	TdT-long [311 K]
Asp396 –Lys494	77.90	57.67
Asp399 –Lys 494	37.32	26.46
Asp492 –Arg393	32.52	5.05
Glu386 –Lys500	0.12	27.59

#### Essential dynamics showed higher flexibility in the distant fingers and index-finger subdomains

**Fig 6:**
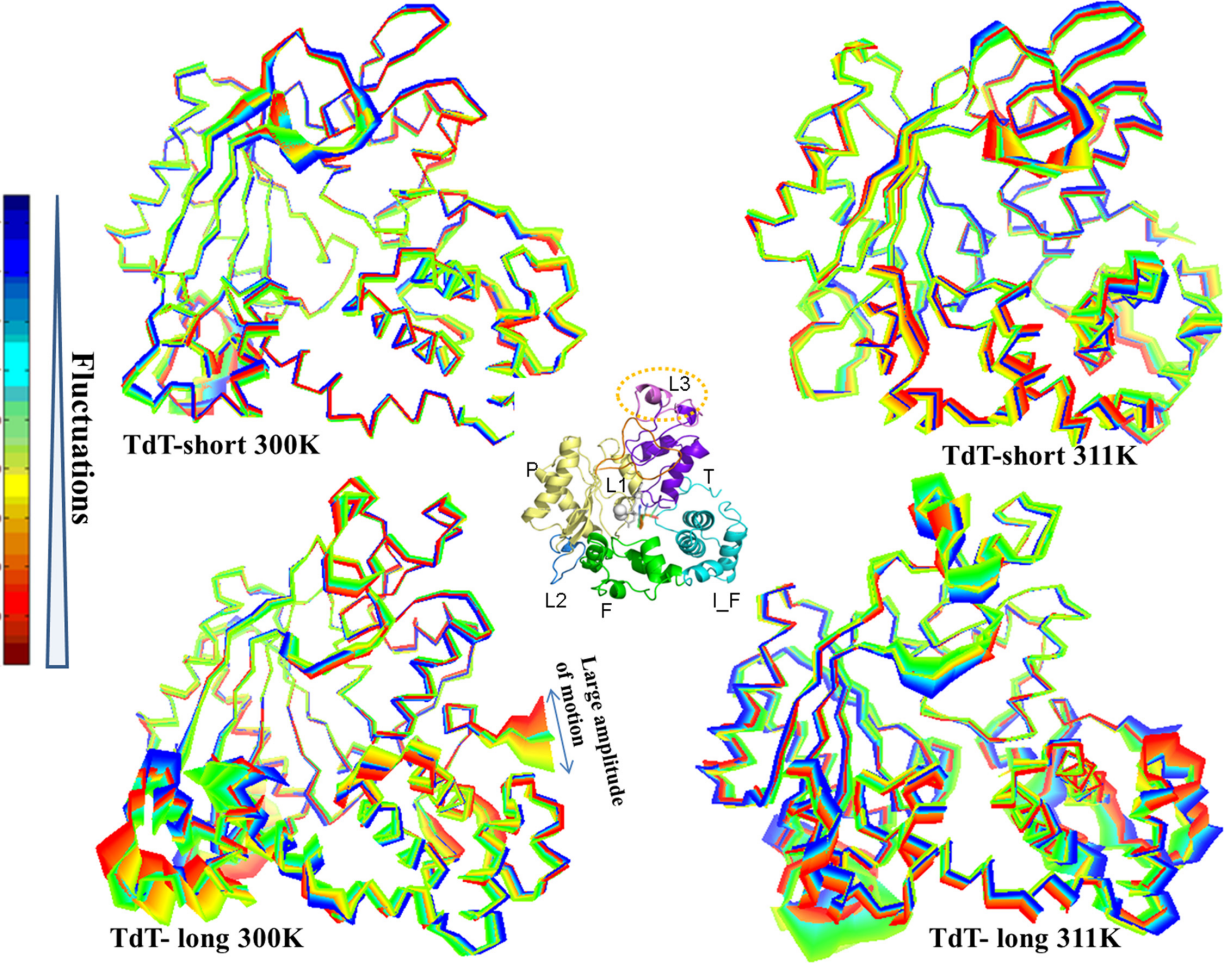
Analysis of global and inter-residue motions in both isoforms. Essential dynamics studies in form of covariance matrices and their resultant PCA were performed at all simulation temperatures but shown here only for 300 K and 311 K. These analyses were performed to characterize the motion of Loop3 and its effect on other regions of TdT. The first two principal components have been analyzed in detail and mapped onto the TdT-short form and TdT-long form three-dimensional structures. Index: Lower frequency fluctuations (blue), higher frequency fluctuations (red), High amplitude -> covering larger space during motion.

Essential dynamics analyses highlighted that the movements of the TdT-short form were considerably reduced than TdT-long form (Fig 6, see details in S1 File) Further, correlation matrices for both isoforms, derived from the covariance matrices (calculated for PCA) at 311 K were compared. A lower frequency of fluctuations in TdT-short isoform was observed (S7 Fig) but registered prominent fluctuations in TdT-long form, especially with respect to the thumb region. A correlated motion between Loop1 and Loop3 was also observed (Fig 5). The movements observed by thumb, index finger and fingers seem to be “claw-like” which might be more pronounced due to the presence of Loop3. The integration of observations from PCA/co-variance and correlation matrices in case of TdT-long isoform, suggests that the presence of the distal Loop3 could provide higher flexibility to the fingers and index-finger regions of the TdT fold; thereby, indirectly enhancing the thermosensitivity of the long isoform.

#### Protein residue network analysis shows compactness of Loop3-Loop1 and loss of interactions in fingers, index-fingers subdomains

**Fig 7:**
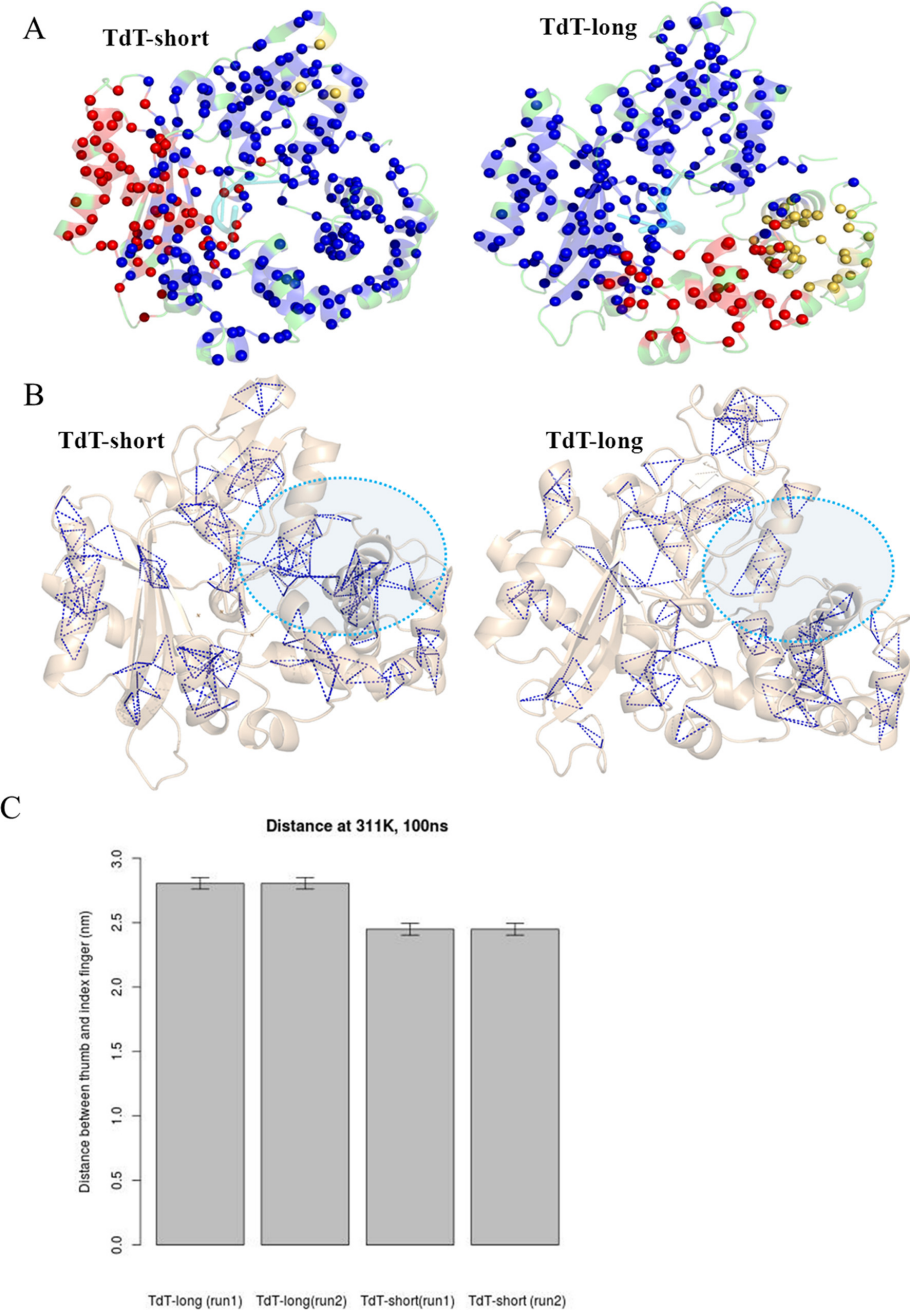
Protein residue network analysis to depict atomic level interactions. a) Formation of clusters in TdT-short form and TdT-long form, b) Unique cliques detected in TdT-short form and TdT-long form. Absence of clique in TdT-long form has been pointed by a cyan coloured dotted circle; c) Distances measured between residues of thumb and index finger for both isoforms (run1 and run2) plotted as bar graph.

Separate residue clusters (connected group of residues in protein residue networks) were formed at the fingers and index-finger subdomains in the shorter isoform compared to a single cluster formed in longer isoform. This, along with the absence of any clique (highly connected region) formation between thumb and index finger, indicated disruption of interactions (Fig 7A and 7B). While the correlated motion of Loop1 and Loop3 (positioned on thumb), along with cluster/clique formation between them indicate their strong connectivity, formation of separate clusters of individual fingers and index fingers indicate loss of stabilizing interactions between thumb and index finger [See details of cliques and cluster formation in S1 File]. This might lead to the increased floppiness of index finger, as observed from the increase in distances between them (Fig 7C).

Also, while comparing the various Loop3 conformers (iTASSER-model, Loopmodel8, Loopmodel22 and Loopmodel23, please see Methods for details), its presence cast "weight" across the index finger and increased the peripheral fluctuations (S9 and S10 Figs). This occurred even in case of reduced interactions with Loop1 as seen in only loopmodel 23 (S10 Fig).

### Role of Loop3 in affecting polymerization rate and specific activity of TdT-long isoform

Following events observed during simulation studies might explain the disorientation of the reaction center and reduced polymerization rate, as well as specific activity of the long TdT isoform.

#### Deformation of catalytic triad

**Fig 8:**
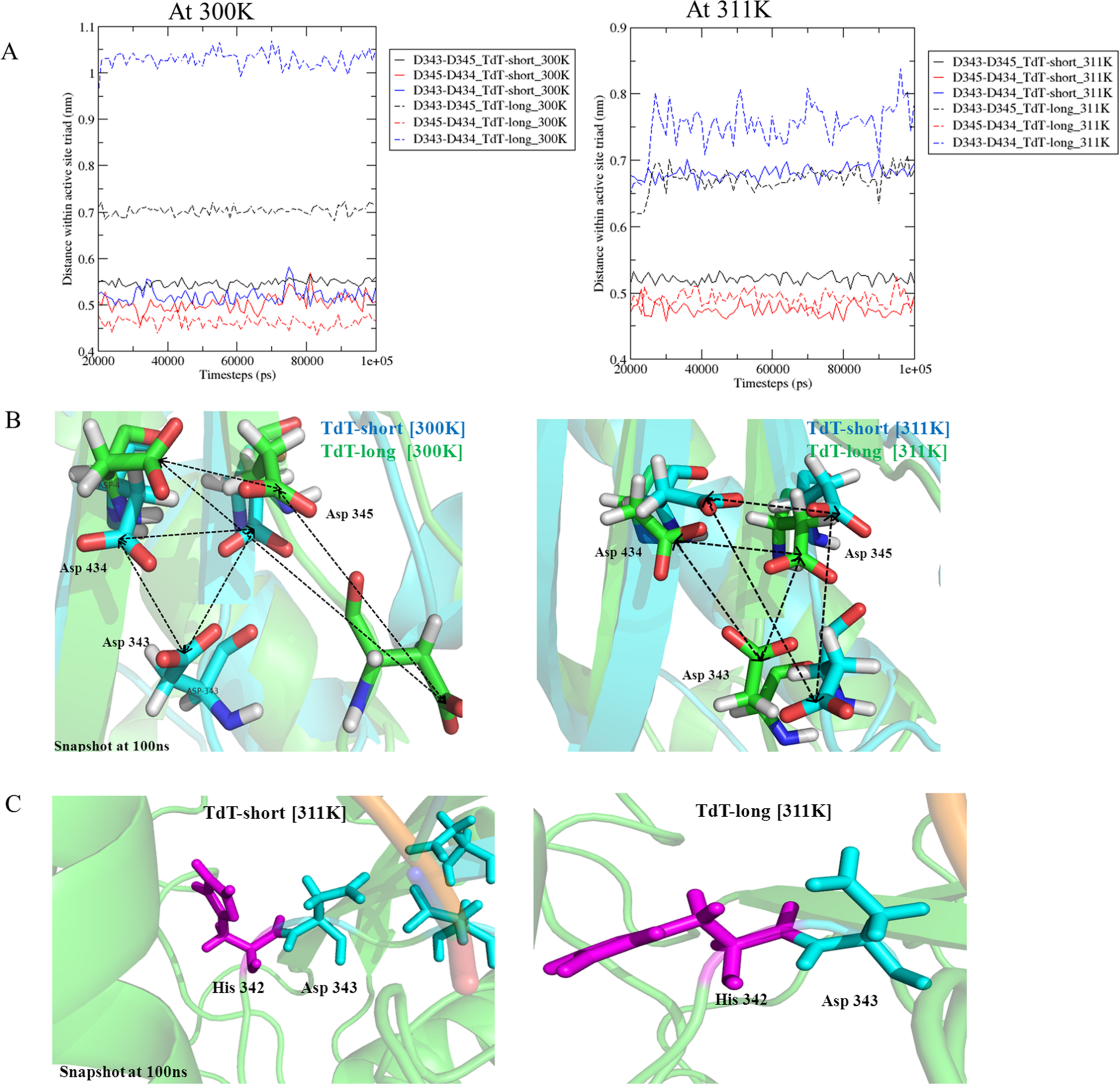
**Effect of insert on the catalytic triad and the neighbouring residues** a) Superposition of active site triad in TdT-short (blue) and TdT-long form (green). Distances between active site triad (Asp343, Asp345 and Asp434) residues in pairwise manner at 300 K and 311 K temperature. Index: TdT-short form marked in complete lines, TdT-long form in dashed lines. b) Snapshots of the deviations in distances within catalytic triad across both isoforms. Asp343-Asp345 pair and Asp343-Asp434 pair show longer distances within them thus indicating distortion of active site triad. TdT-long form showed deviation in distances for two out of three pairs (D343-D434, D343-D345) at even physiological temperature (300 K). c) Orientation of His342 in TdT-short form (left) and TdT-long isoform (right) during simulations at 311 K temperature. His342 side chain is flipped out in TdT-long form for majority of the 100 ns of simulation time.

TdT follows “two-divalent-metal ion” mechanism [45], similar to other known polymerases and contains three Aspartate residues (Asp343, Asp345 and Asp434) in the palm region that forms the active site. While the distance between the active site triad residues were maintained in TdT-short form during simulations at different temperatures, TdT-long form showed deviation in distances for two out of three pairs (Catalytic pair a) D343-D434: 5.19±0.15 Å (TdT-short_300 K) 10.2±0.17 Å (TdT-long_300 K); b) D343-D345: 5.4±0.07 Å (TdT-short_300 K) 7.03±0.09 Å (TdT-long_300 K)) even at physiological temperature (300 K) (S11 Fig). This showed that the active site triad is slightly deformed in TdT-long isoform, which could contribute to decrease in activity of the enzyme (Fig 8B).

#### Orientation changes in residues proximal to the active site altered the geometric arrangement for the polymerization reaction in TdT-long isoform

Residues, which are proximal to the catalytic triad (active site), *viz* His342 and Trp450, participate to create a favorable reaction center. Previous studies showcased the effect of subtle side chain rearrangements prior to the catalytic chemistry [41]. His342, in palm subdomain has been implicated as crucial for template-independent activity of TdT and Pol μ [46–48] and also has been involved in the release of metal ion and pyrophosphate (Ppi), after the catalysis, by performing simple side chain rotation [22]. In our simulations at 311 K, distances measured between His342 and its neighboring Asp343 showed higher deviations for TdT-short form (mean distance for run1: 9.05±1.01 Å, run2:7.54±1.39 Å), signifying that His342 flips back and forth to aid in product release. On the other hand, distances in TdT-long form were observed to be more stabilized (mean distance for run1: 6.56±0.83 Å, run2: 6.99±0.97 Å). Analysis of MD simulation snapshots showed that His342 had flipped away by side chain rotation from Asp343 (Fig 8C). Thus, the disoriented nature of His342 from the reaction center might lead to lower polymerization in TdT-long form (2–3 folds lower than TdT-short form). Another residue Trp450, which is thought to have stacking interactions with DNA and help orient the last nucleoside of DNA, also showed different orientation (unfavorable for stacking) in the TdT-long isoform (S12 Fig).

#### Shifting of DNA primer in TdT-long isoform disrupted the reaction center

**Fig 9:**
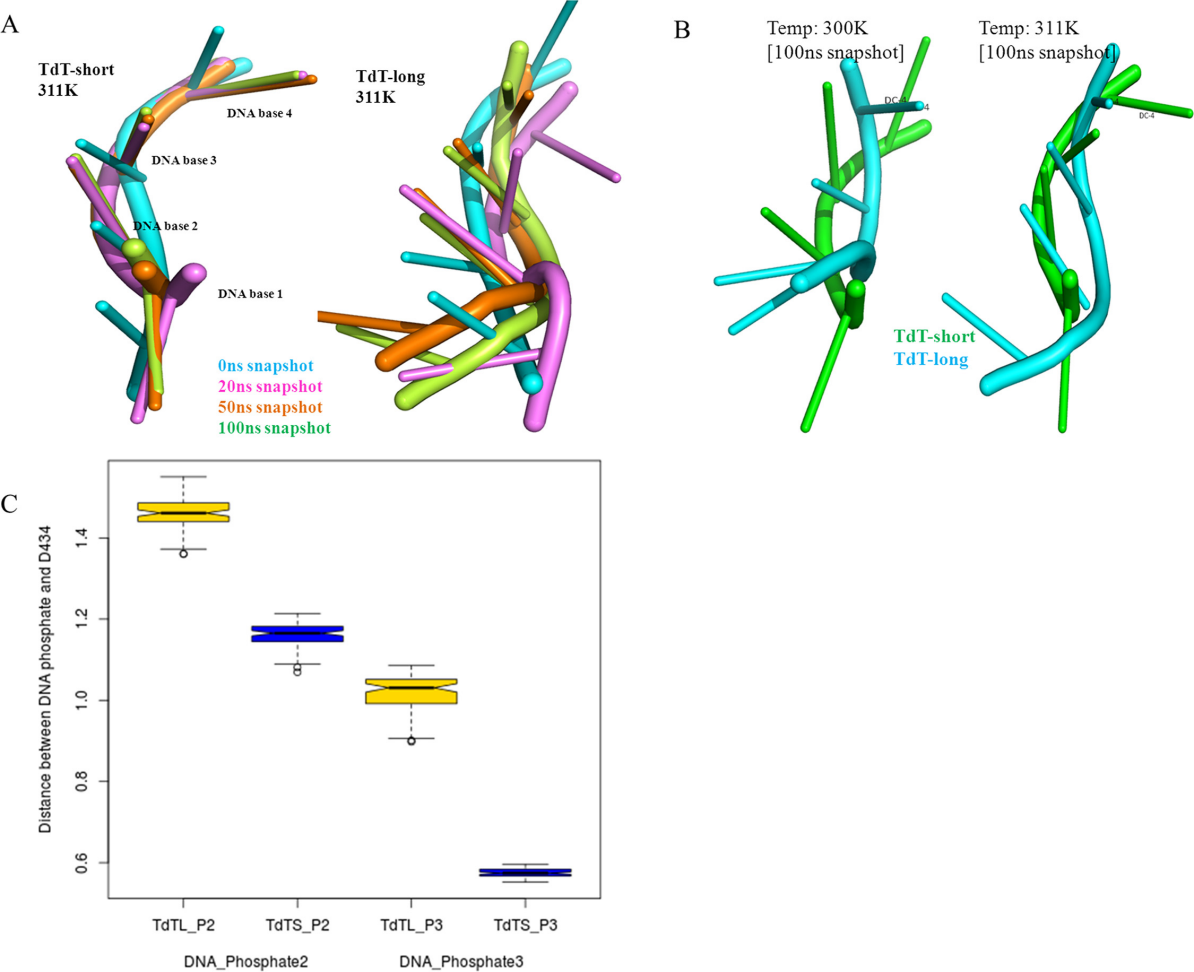
**Parameters to measure shifting of DNA primer in TdT-long isoform** a) Superposed DNA primers at different instances during simulation (at 0, 20, 50 and 100 ns snapshots) for TdT-short form and TdT-long form at 311 K, b) Orientation of the bases and backbone of DNA primer at final snapshot of different temperatures for TdT-short form (green) and TdT-long form (cyan). c) Boxplot of distances measured between DNA phosphates 2 and 3 with active site Asp434 between TdT-short and TdT-long form at 311 K. The extent of variation in TdT-long form quantifies the drifting motion in the DNA primer.

Widening of the active site cavity may affect the proper positioning of the DNA primer which is crucial for the effective catalytic activity of TdT [See details in S1 File]. In our simulations, we observed the DNA primer to have drifted from the active site in TdT-long form at its inactivation temperature of 311 K, unlike TdT-short form where DNA maintains its position in formation of the pre-catalytic geometry (Fig 9A). At the end of 100 ns simulations in TdT-long form (for 300 K & 311 K), while DNA backbone was distorted and bent away from the active site, the bases were bent towards the active site as evident over the simulation period (Fig 9B). Distances between second and third phosphates in the DNA primer, with respect to the active site residue Asp434, showed drifting and rotating motion of the DNA primer in the longer isoform (Fig 9C). Although the 3’OH of the terminal base in DNA (especially at the reaction site) was observed to be proximal to the active site in TdT-long form, the change in orientation of DNA backbone might affect the geometry of the reaction center, thus leading to reduction in polymerization rate.

Docking of DNA and interacting partners of TdT (*viz*. PCNA and TdIF1) onto the TdT-long isoform suggested that there is no hindrance due to the presence of Loop3 [See Methods & Results in S1 File].

## Discussion

TdT belongs to the Pol X DNA polymerases family and exists in two isoforms, longer (TdT-long) and shorter (TdT-short). TdT-long isoform has a twenty residues insert (Loop3) and shows reduced polymerization efficiency as well as increased thermosensitivity compared to TdT-short isoform. In this study, we have tried to explain the structural effects caused by the presence of Loop3, located distal from the active site, on the TdT-long isoform. We have identified conformational changes in the regions distal to the insert position which might indirectly affect the activity and stability of the enzyme.

TdT is a template-independent, low-fidelity polymerase and would not require any subdomain motion to adjust to the incoming nucleotide. However, there may be side chain adjustments in achieving the optimal electrostatics and geometry of the active site prior to the enzyme catalysis [43,49]. Essentially, the positioning of catalytic triad, metal ions, incoming dNTP and the terminal nucleotide of primer are essential for the efficient polymerization process. As most of these changes occur through small conformational changes, tracing the phenomenon becomes more difficult from a static perspective of crystal structures. Hence, a molecular dynamics approach has been employed here to elucidate the system-level and residue-level changes occurring in TdT isoforms at various temperature ranges for 100 ns. It is known that heuristic approximations and inabilities to sample entire conformational space are some of the limitations of molecular dynamics simulations and we have performed duplicate runs for TdT isoforms to address this issue.

Similar length insert (Loop3) is also observed in the thumb subdomain of pathogenic pol β, which interacts with the template and its deletion caused higher catalytic efficiency [6]. This observation is similar to TdT where the smaller isoform [without Loop3] has higher activity. According to our simulation studies, TdT-long form is also more structurally dynamic than its shorter counterpart (Fig 2A). TdT-long form, as compared to TdT-short form, showed varied intensity of fluctuations in the subdomains at physiological (300 K) and inactivation temperatures (311 K), which might be due to the inclusion of Loop3 (Fig 3, S1 and S2 Movies). This insert is located distal from the active site and thus cannot directly affect the catalysis. However, as observed in molecular dynamics simulations, the Loop3 is able to mediate conformational changes through the intermediate Loop1.

Loop1 is involved in template-independent synthesis in Pol μ and TdT and is present in the palm region of the hand-like polymerase structure, Recent studies have shown that mutations carried out in Loop1 led to altered catalytic properties [44]. Flexibility of Loop1 has been implied to allow the binding of different substrates through various Loop1 conformations and structural evidence suggests that the Loop1 might behave as a pseudo-template and aid in stabilizing the primer terminus [44,50]. Our results indicate that Loop3 interacts with Loop 1 and increases its restraints, thus further limiting the flexibility of Loop1. Loop1 is observed to be closer to the active site cavity and possess stronger interactions with neighboring residues in the presence of Loop3 in TdT structure from the correlation analysis and distance measurements (Figs 4, 5 and 6, Table 4). Hence, part of the palm and thumb subdomains which possess the Loop1 and Loop3, respectively, are bound together (as also seen by correlated movements in section C, D of Fig 5).

**Fig 10:**
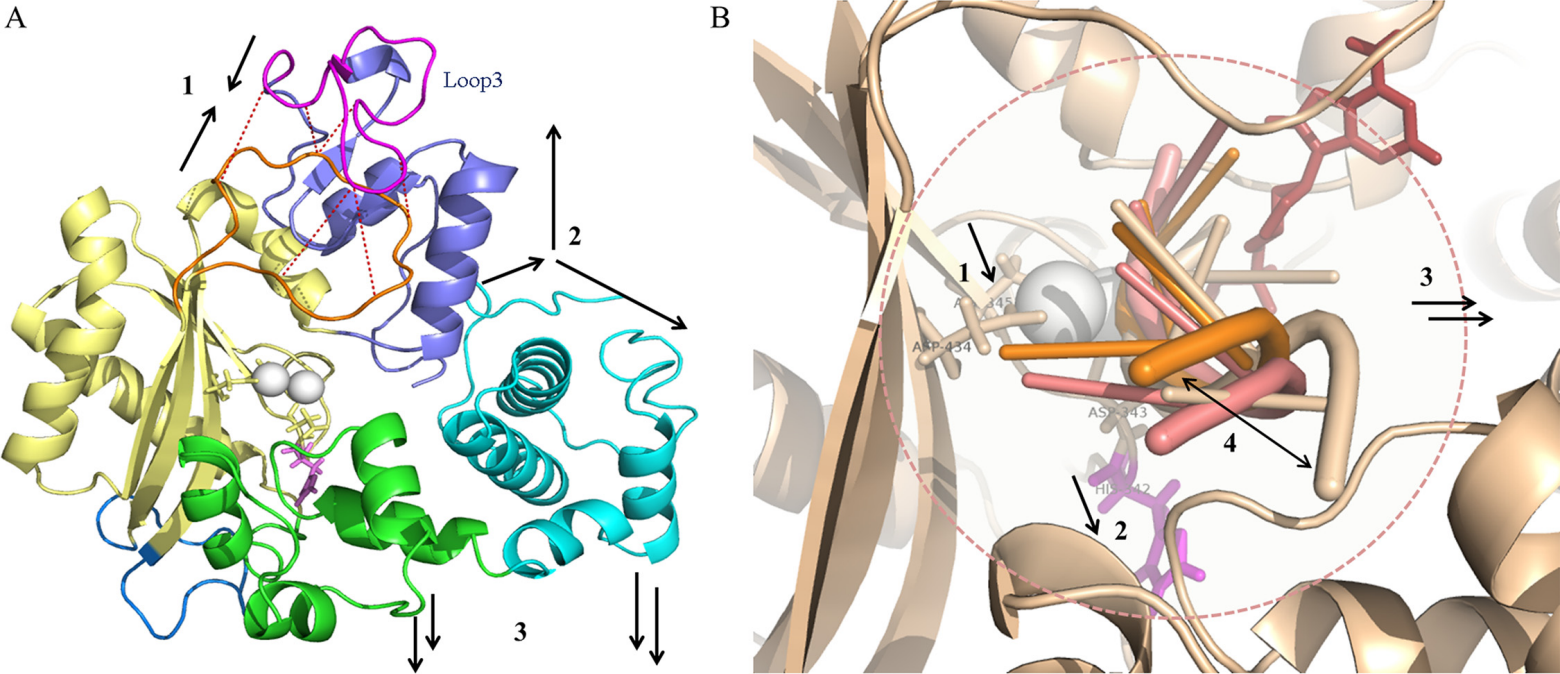
Schematic illustrating the major conformational changes observed during molecular dynamics mapped onto TdT-long isoform. a) Events leading to increasing thermosensitivity: 1.Hydrogen and electrostatic interactions bind Loop1 and Loop3, 2. Weakening of interaction between thumb and index finger, 3) Increase in fluctuations in index finger and finger subdomains. b) Events leading to decrease in polymerization activity: 1. Deformation of active site triad, 2. Flipping away of His342 side chain, 3. Widening of active site cavity and 4. Shifting of DNA primer.

Regions like fingers, thumb and index finger subdomains showed higher fluctuations in TdT-long form (also observed consistently among all the top Loop3 conformers of TdT-long form) (Figs 2 and 3 and Tables 2 and 3). This phenomenon was observed along with loss of connections between thumb and index finger, as shown by enhanced flexibility with respect to TdT-short isoform in the PCA, correlation matrices and network properties analyses (Figs 5, 6 and 7). Further, the heightened fluctuations (floppiness) in the index-finger are propagated to the fingers subdomain as evident by the increased amplitude bandwidth in PCA mapping (marked by arrow in Fig 6). It has been shown previously in other studies that molecular flexibility, while important for substrate binding and catalysis, needs to be balanced with stability of the protein. Thus, the increased floppy nature of the peripheral regions along, with overall reduction of intradomain interactions (as reflected by less interactions between thumb-palm cluster and index finger subdomain), may be responsible for the enhanced thermosensitivity of the longer isoform (Fig 10A).

Fluctuations in distances between the catalytic residues, as observed in the simulations of long isoform lead to changes in catalytic triad geometry while, restricted flipped-back motion of neighbouring His342 side chain rearrangement might hinder the easy release of the products. Further analyses performed indicate that widening of active site cavity (S1 File and Fig 8, S17 Fig) could further lead to dislocation of the dNTP and DNA primer, from the preferred geometry. The position of the nucleotide was further observed to have shifted during the course of simulations and also rotated facing the active site (Fig 9). Overall, all these events might be a result of allosteric changes occurring within the protein due to insertion of Loop3 in TdT-long isoform. These might be cumulatively responsible in disrupting the reaction center geometry in TdT-long form structures (especially at higher temperatures i.e. inactivation temperatures) in its pre-catalytic state and thus, does not permit the DNA strand to associate efficiently. This, in turn, would reduce the speed in extending the single-stranded DNA polymer, thus reducing the polymerization rate and activity of TdT-long isoform (Fig 10B). Hence, these results provide a structural rationale for the reduced polymerization rate and increased thermosensitivity as observed in biochemical assays in mouse TdT-long isoform by Boule and co-workers [10]. Also, we observed that the inclusion of the insert on the surface of the protein did not cause any hindrance to protein-DNA or protein-protein interactions essential for its regulation.

Template-dependent DNA polymerases undergo major structural rearrangements during dNTP binding and consecutive catalysis. The two deviations from this norm are Pol μ, Pol λ and TdT which always form a closed form and thumb, Loop1 and few side chain rearrangements are the only movements visible during dNTP binding. In such a scenario, presence of an insert causing structural changes across TdT isoform is very interesting and provides insight in protein engineering of TdT during drug discovery experiments. Similar structural and simulation studies have been performed elsewhere, wherein a two-residue insert in a distal loop did not affect the global fold of a SH2 domain, but affected its catalytic activity [51]; and also in the case of leucyl-tRNA synthetase where the insert modulated the editing activity of the enzyme [52].

## Conclusion

Modeling of insertions and deletions is quite challenging due to the inherent difficulty in modeling loop conformations and unpredictable cascade of events which can disrupt the stability of protein structure [53]. In this study, we have modeled twenty residue insertion (loop3), present in the murine isoforms of template-independent TdT. This was then followed by structural validation and molecular dynamics to study the structural effects within the enzyme, to explain the modulation of stability and activity of the enzyme, due to this Loop3 insertion. We observed that in the short isoform TdT, Loop1 overhangs the active site and contributes in maintaining the relevant residues in the conformation necessary for catalysis. In the long form, the inserted Loop3 is on the other side of Loop1 and engages contact with Loop1 during simulations. Our simulation studies suggest that these new contacts render Loop1 and Loop3 as a subdomain, but result in loss of contacts with index finger domain and ultimately the index-finger and finger subdomains to become structurally more floppy. The resultant enhanced fluctuations, in turn, results in the orientation changes of the catalytic site residues and a larger cavity for the polynucleotide product, leading to the observed functional and biochemical differences. In summary, a structural rationale has been presented to explain the different biochemical properties in the presence and absence of Loop3 of TdT. This will further help in designing experiments in other members of Pol X family of DNA polymerases. This study provides an opportunity to understand the structural effects of length variations in protein families (in the form of insertions and deletions) on the activity and stability of isoforms.

## Supporting Information

S1 Fig Validation of models of Loop3 conformers of TdT-long isoform. a) Modeling of Loop3 in TdT-short form (cyan) by iTASSER (green) and PRODAT loop library of SYBYL7.2 (Tripos package) (top scoring models in magenta (loopmodel 8), yellow (loopmodel 12), cream (loopmodel 22) and grey (loopmodel 23)). iTASSER model has been chosen due to presence of helical part and due to highest scoring provided by structural validation tools. b) Electrostatic surface of TdT-long form. Positive charges (blue) conferred by lysines shown with dashed circle. Model validation performed by c) PROSA and d) HARMONY.
(TIF)

S2 Fig Fluctuations registered between both isoforms at different simulation temperatures. a) Root Mean Square Fluctuations (RMSF) of TdT-short form and TdT-long form in its free and ligand bound state. TdT-short form maintained similar RMSF in both free and ligand bound form but TdT-long form showed higher RMSF in free form than in ligand bound form. Index: TdT-short form (black), TdT-long form (red), free enzyme (complete lines) and ligand bound enzymes (dashed lines). b) RMSF of TdT-short form at all simulation temperatures. Index: 298 K (black), 300 K (red), 311 K (green) and 318 K (blue).
(TIF)

S3 Fig Essential dynamics depicted as PCA for the mutated TdT-long form. Absence of electrostatic interactions (or salt bridges) in mutated TdT-long form, within Loop3 and Loop1, caused enhanced fluctuations in each of these regions. This is also demonstrated in the covariance/PCA values which were mapped onto the mutated TdT-long form structure along with the DCCM plots. a) Projection of first two dominant modes of PCA, b) correlation matrices of mutated TdT-long form trajectories at 300 K. Less amplitude fluctuations observed by PCA plot in the mutated form as compared to wild-type form (Fig 6)
(TIF)

S4 Fig Fluctuations compared in wild type and mutated TdT-long form. a) RMSF of all C-alpha atoms of wild-type (red) and mutated (magenta) of TdT-long form at 300 K. Mutation of lysines to alanine in Loop3led to increase in fluctuations in Loop3 and Loop1. b) Bar graph of RMSF of Loop1 from TdT-short and TdT-long isoforms (run1 and run2). Loop1 had lower RMSF in TdT-long form due to the presence and electrostatic binding with Loop3. Comparison of fluctuations (RMSF values) across Loop1 showed lower values in TdT-short form with respect to long form, which also substantiates the uninhibited fluctuations of Loop1 in the shorter isoform.
(TIF)

S5 Fig Fixation of Loop1 region by its surrounding residues. Distances observed between of residues in Loop1 and its neighborhood residues which indicates stronger fixation of Loop1 in TdT-long form (red) than TdT-short form (black). a) Asp399 –Lys403, b) Phe401-His475 and c) Ser392-Asp473
(TIF)

S6 Fig Mapping of covariance matrices and PCA (top two dominant modes) onto TdT-long form structure. Simulations extended till 300 ns at 311 K but similar results as shown in Fig 6 obtained.
(TIF)

S7 Fig **Increase in subdomain motions in TdT-long isoform as observed during simulations** a) Comparison of correlation matrices of TdT-short and TdT-long at 311 K. Positive values (red spectrum) indicate correlated motion; negative values (blue spectrum) indicate anti-correlated motions between sub-domains. Higher correlations observed in TdT-long form than in TdT-short form. b) Arrow diagram of simulations at 311 K based on covariance matrices shows that TdT-short (left) does not undergo much fluctuations but TdT-long (right) has maximum motions in index finger, fingers and Loop3 regions
(TIF)

S8 Fig Unique edges of cliques mapped onto TdT-short form and TdT-long isoform structure at 100 ns. Common edges to form forms coloured in red, unique to only short form in blue and unique to only long form coloured in magenta. Most of the unique edges of cliques of TdT-long is located near thumb and Loop1
(TIF)

S9 Fig Similar trends of perturbation observed across different Loop3 conformers. a) PCA from essential dynamics studies of simulations carried out at 311 K mapped onto the different loop conformers of TdT-long form (iTASSER loopmodel, Loopmodel8, Loopmodel 22) and TdT-short isoform (for comparison); b) Distances observed between of residues in Loop1 and Loop3 among various loopmodels of TdT-long form.
(TIF)

S10 Fig Correlation matrices for Loop3 conformers of TdT-long form when simulated at 311 K. The top-ranking loop conformers of TdT-long isoform are a) iTASSER loopmodel, b) Loopmodel 8, c) Loopmodel 22 and d) Loopmodel 23 (from PRODAT library of SYBYL, Tripos package). Positive values (red spectrum) indicate correlated motion; negative values (blue spectrum) indicate anti-correlated motions between sub-domains. Sub-domains and loops of TdT has been marked on X-axis and Y-axis in different colours as: index finger or “I_F” (blue), fingers or “F” (green), palm or “P” (yellow), thumb or “T” (violet), Loop1 or “L1” (orange), Loop2 or “L2” (blue) and Loop3 or “L3” (magenta). Similar colour coded TdT-long structure has been provided at top left corner for ease in understanding.
(TIF)

S11 Fig Distances between each pair of catalytic triad illustrated for TdT isoforms at 300 K and 311 K. Snapshots of Asp343-Asp434 pair show longer distances within them in TdT-long isoform thus indicating distortion of active site triad.
(TIF)

S12 Fig Effect on surrounding residues in catalytic site. Distance of Trp450 from active site (D434) [upper left panel] and last nucleoside of DNA primer [lower left panel]. Orientation of Trp450 in short and long isoform of TdT [upper and lower right panel respectively].
(TIF)

S13 Fig Poses of DNA primer docked onto TdT-long isoform by AUTODOCK. Lack of DNA primer docked to the Loop3 despite being high in positive charges depicts that the presence of insert did not alter the binding of DNA primer in the active site cavity. [Details referred in S1 File]
(TIF)

S14 Fig Illustration of TdT-long docked with its interacting partners. a) TdIF1 and b) PCNA near the thumb region. Binding at similar positions behind the thumb region support the theory that these proteins may competitively bind in order to regulate TdT. [Details referred in S1 File]
(TIF)

S15 Fig **Effect of co-existing domain in TdT** a) model of TdT-long with BRCT domain, b) PCA of simulation trajectories at 300 K mapped onto the model. [Details referred in S1 File]
(TIF)

S16 Fig Docking poses (by GRAMM-X method) of TdT-long form in putative homodimer formation. Loop3though having mostly hydrophobic residues and lying on surface is not found to lie in the dimer interface and hence does not make TdT-long form prone to homodimer formation. [Details referred in S1 File]
(TIF)

S17 Fig Widening of active site cavity in TdT-long isoform. a, b)Surface representation of final snapshot of TdT-short and TdT-long form after simulations at 311 K temperature. Active site cavity/pore size of TdT-long form is wider than short form as circled in the figure. c)Pore shape and mapping of pore (red-green) onto TdT-short isoform and d) TdT-long isoform. Longer isoform has an expanded cavity/pore as compared to shorter isoform, e) Hydrogen bonds and f) solvent accessible surface area of the active site cavity for TdT-short (complete lines) and TdT-long isoforms (dashed lines) at physiological temperature of 300 K (black) and inactivation temperature of long form at 311 K(red).
(TIF)

S1 File (Supplementary text/Methods/Results) Analysis of widening of active site cavity observed in simulations. Interaction of TdT with incoming DNA primer, BRCT domain and regulatory proteins and effect of presence of twenty residue insertion.
(DOC)

S1 Movie Illustration of TdT-long form dynamics in 100 ns molecular dynamics simulations performed. High fluctuations in Loop3, fingers and index fingers regions as well as in DNA primer is seen while lower fluctuations of Loop1 as compared to TdT-short form are noted. Mg2+ ions and ddATP not included in the movie.
(MPG)

S2 Movie Illustration of TdT-short form dynamics in 100 ns molecular dynamics simulations performed. High fluctuations of Loop1 and Loop2 observed while rest of structure and DNA primer showed lesser fluctuations when compared to TdT-long form.
(MPG)
