# Supplementary figures and images for: Molecular Dynamics Simulations and Structural Analysis to Decipher Functional Impact of a Twenty Residue Insert in the Ternary Complex of Mus musculus TdT Isoform
Source: PLoS One. 2016 Jun 16;11(6):e0157286. doi: 10.1371/journal.pone.0157286 (PMC4911049; doi:10.1371/journal.pone.0157286)

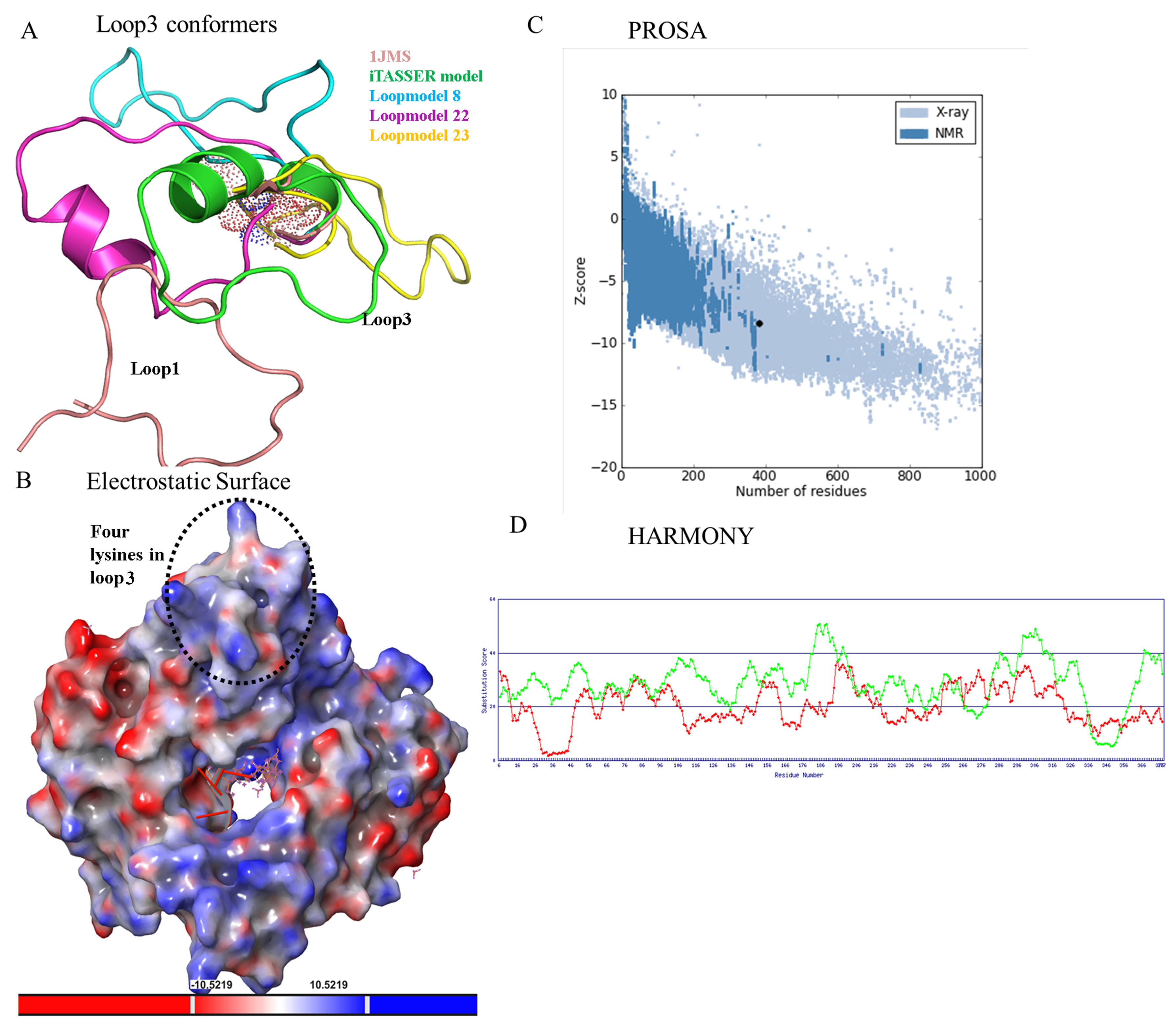

Supplement: S1 Fig — a) Modeling of Loop3 in TdT-short form (cyan) by iTASSER (green) and PRODAT loop library of SYBYL7.2 (Tripos package) (top scoring models in magenta (loopmodel 8), yellow (loopmodel 12), cream (loopmodel 22) and grey (loopmodel 23)). iTASSER model has been chosen due to presence of helical part and due to highest scoring provided by structural validation tools. b) Electrostatic surface of TdT-long form. Positive charges (blue) conferred by lysines shown with dashed circle. Model validation performed by c) PROSA and d) HARMONY. (TIF) [file pone.0157286.s001.tif]

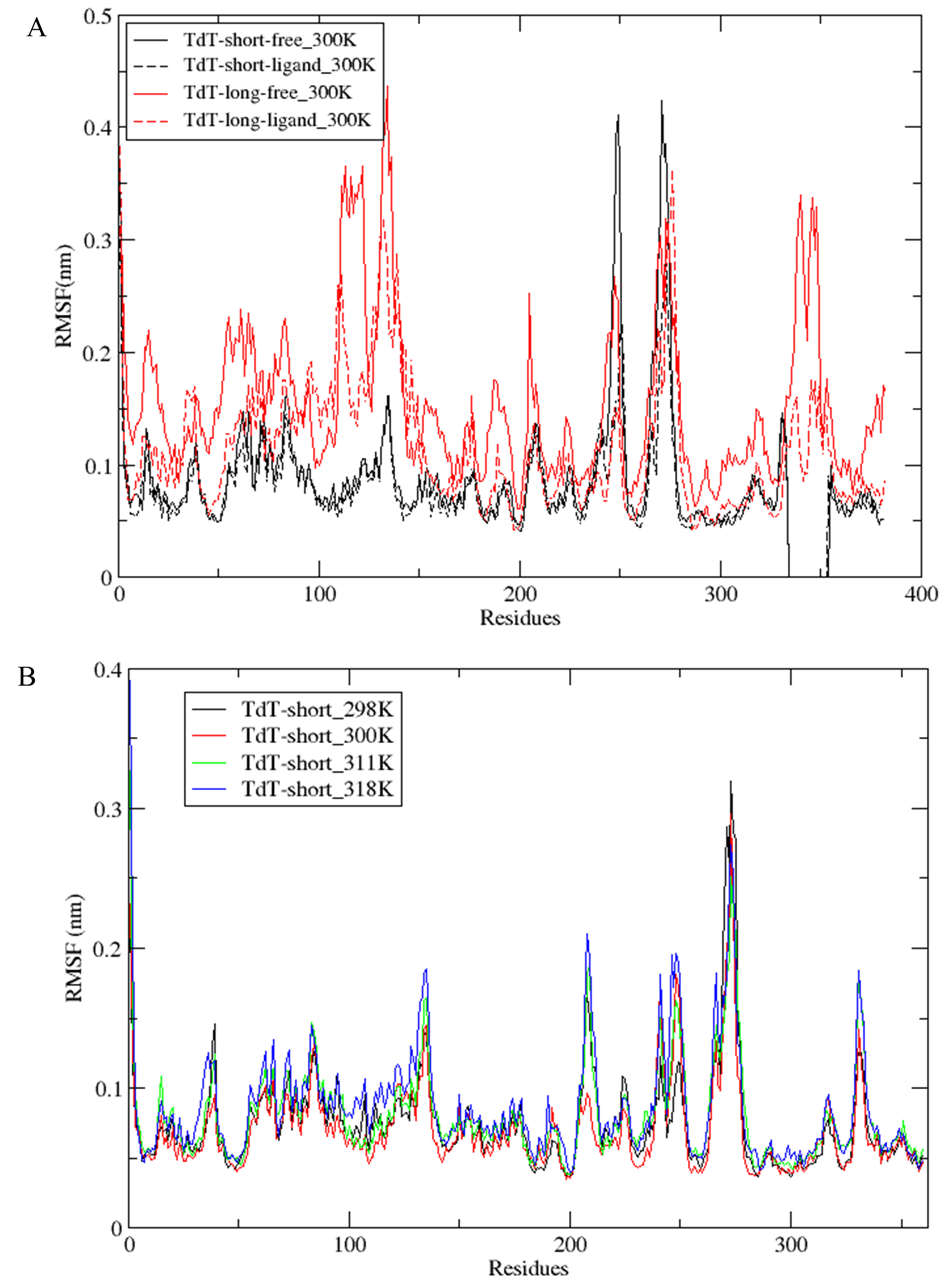

Supplement: S2 Fig — a) Root Mean Square Fluctuations (RMSF) of TdT-short form and TdT-long form in its free and ligand bound state. TdT-short form maintained similar RMSF in both free and ligand bound form but TdT-long form showed higher RMSF in free form than in ligand bound form. Index: TdT-short form (black), TdT-long form (red), free enzyme (complete lines) and ligand bound enzymes (dashed lines). b) RMSF of TdT-short form at all simulation temperatures. Index: 298 K (black), 300 K (red), 311 K (green) and 318 K (blue). (TIF) [file pone.0157286.s002.tif]

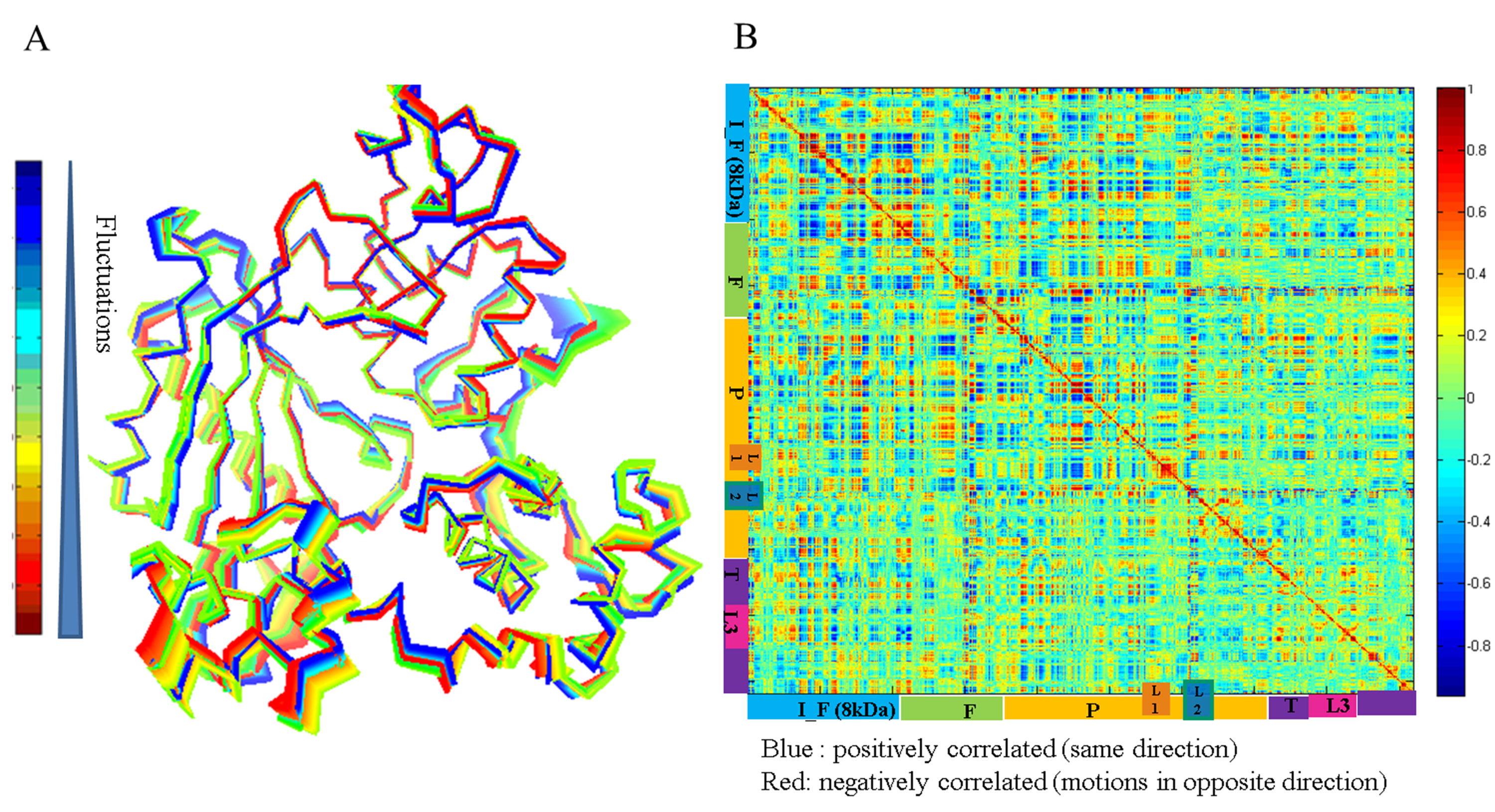

Supplement: S3 Fig — Absence of electrostatic interactions (or salt bridges) in mutated TdT-long form, within Loop3 and Loop1, caused enhanced fluctuations in each of these regions. This is also demonstrated in the covariance/PCA values which were mapped onto the mutated TdT-long form structure along with the DCCM plots. a) Projection of first two dominant modes of PCA, b) correlation matrices of mutated TdT-long form trajectories at 300 K. Less amplitude fluctuations observed by PCA plot in the mutated form as compared to wild-type form (Fig 6) (TIF) [file pone.0157286.s003.tif]

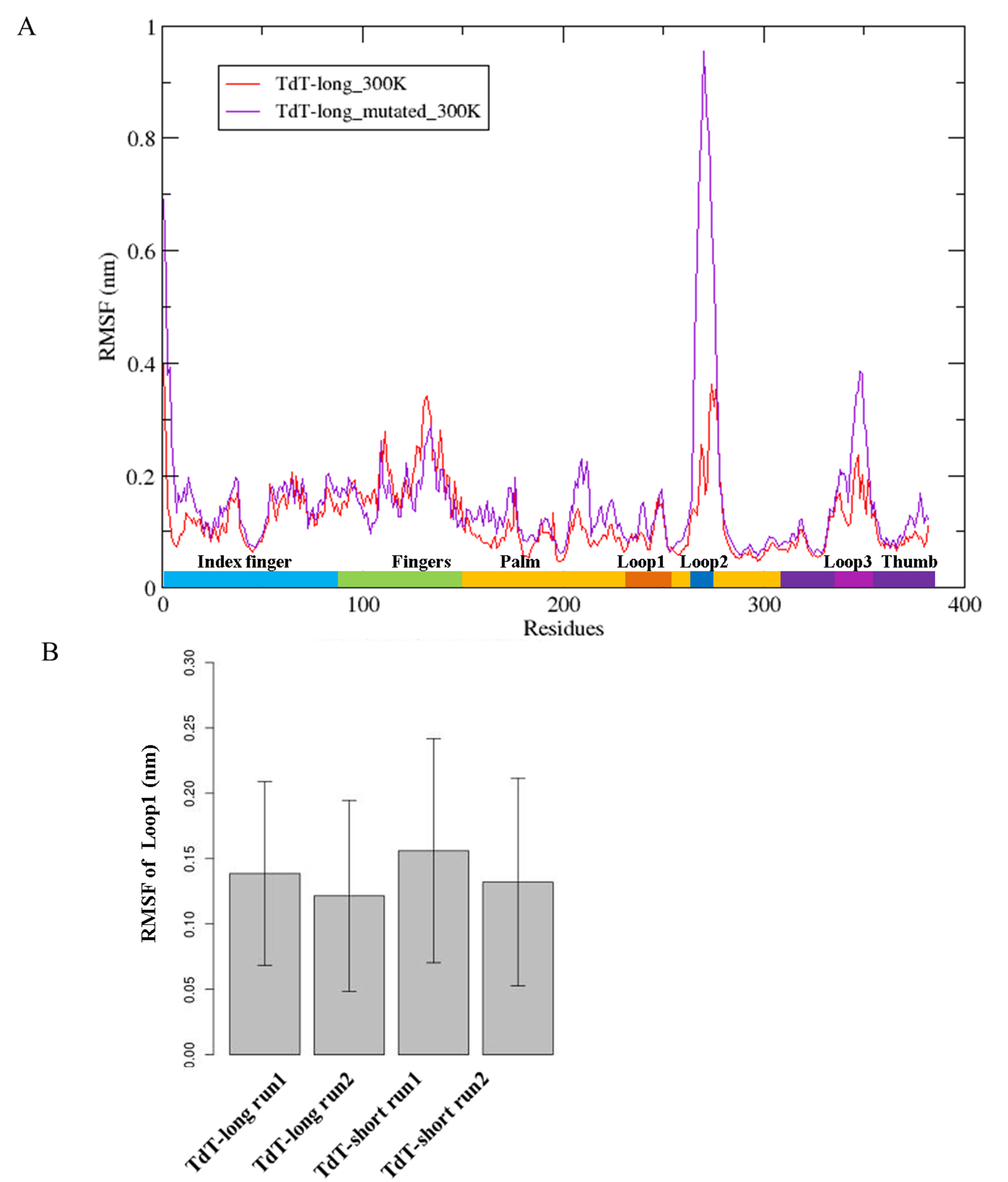

Supplement: S4 Fig — a) RMSF of all C-alpha atoms of wild-type (red) and mutated (magenta) of TdT-long form at 300 K. Mutation of lysines to alanine in Loop3led to increase in fluctuations in Loop3 and Loop1. b) Bar graph of RMSF of Loop1 from TdT-short and TdT-long isoforms (run1 and run2). Loop1 had lower RMSF in TdT-long form due to the presence and electrostatic binding with Loop3. Comparison of fluctuations (RMSF values) across Loop1 showed lower values in TdT-short form with respect to long form, which also substantiates the uninhibited fluctuations of Loop1 in the shorter isoform. (TIF) [file pone.0157286.s004.tif]

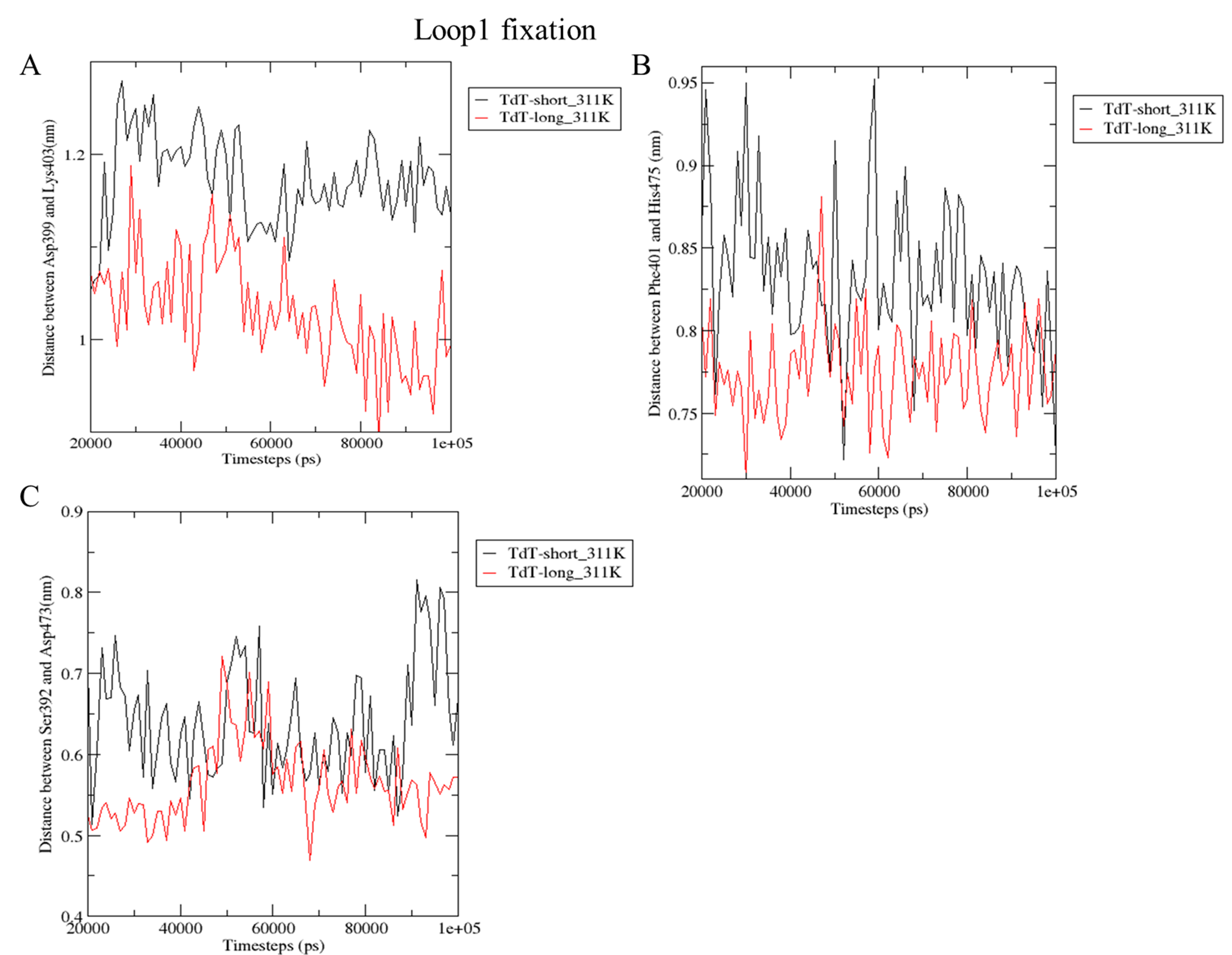

Supplement: S5 Fig — Distances observed between of residues in Loop1 and its neighborhood residues which indicates stronger fixation of Loop1 in TdT-long form (red) than TdT-short form (black). a) Asp399 –Lys403, b) Phe401-His475 and c) Ser392-Asp473 (TIF) [file pone.0157286.s005.tif]

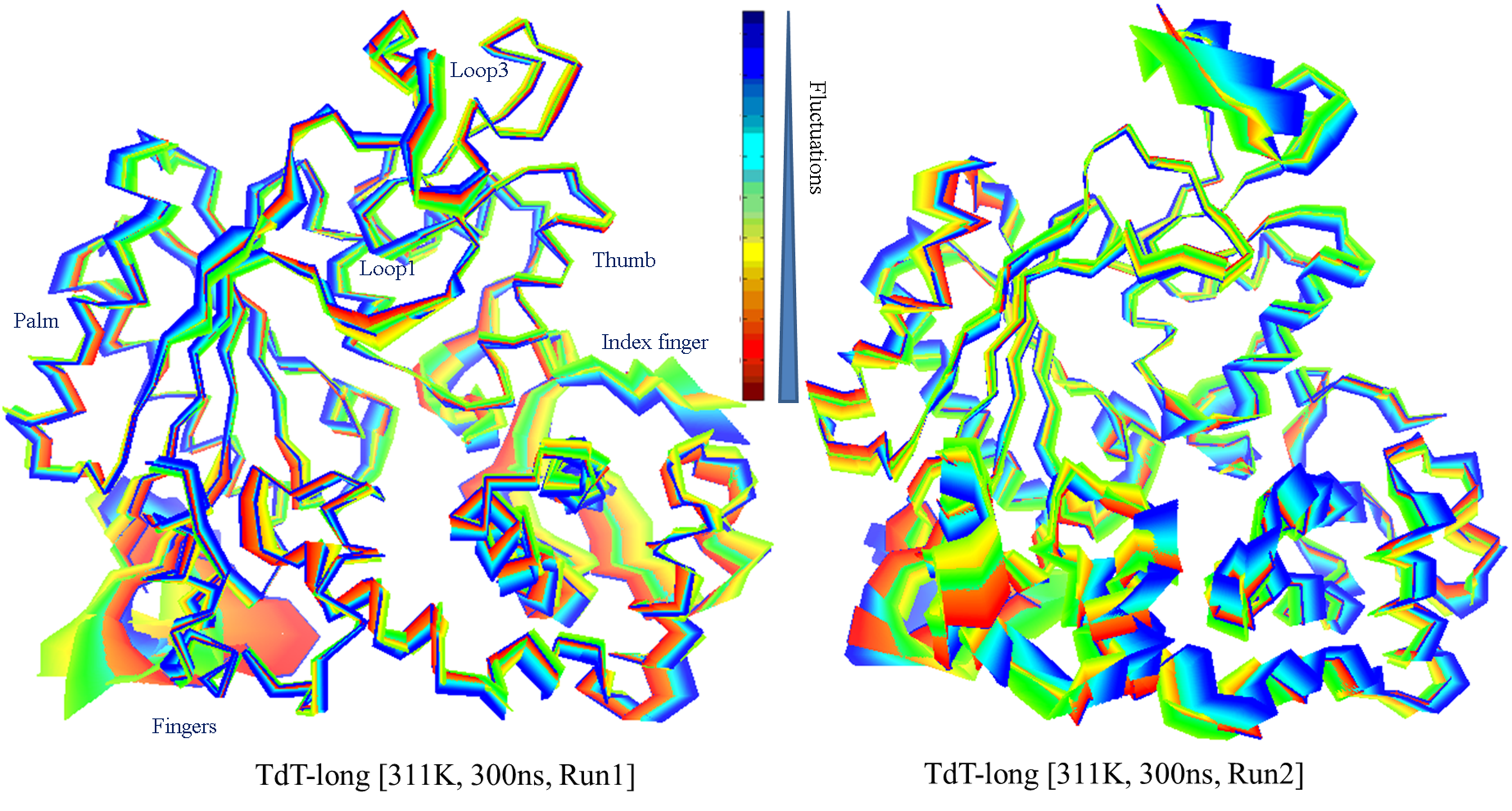

Supplement: S6 Fig — Simulations extended till 300 ns at 311 K but similar results as shown in Fig 6 obtained. (TIF) [file pone.0157286.s006.tif]

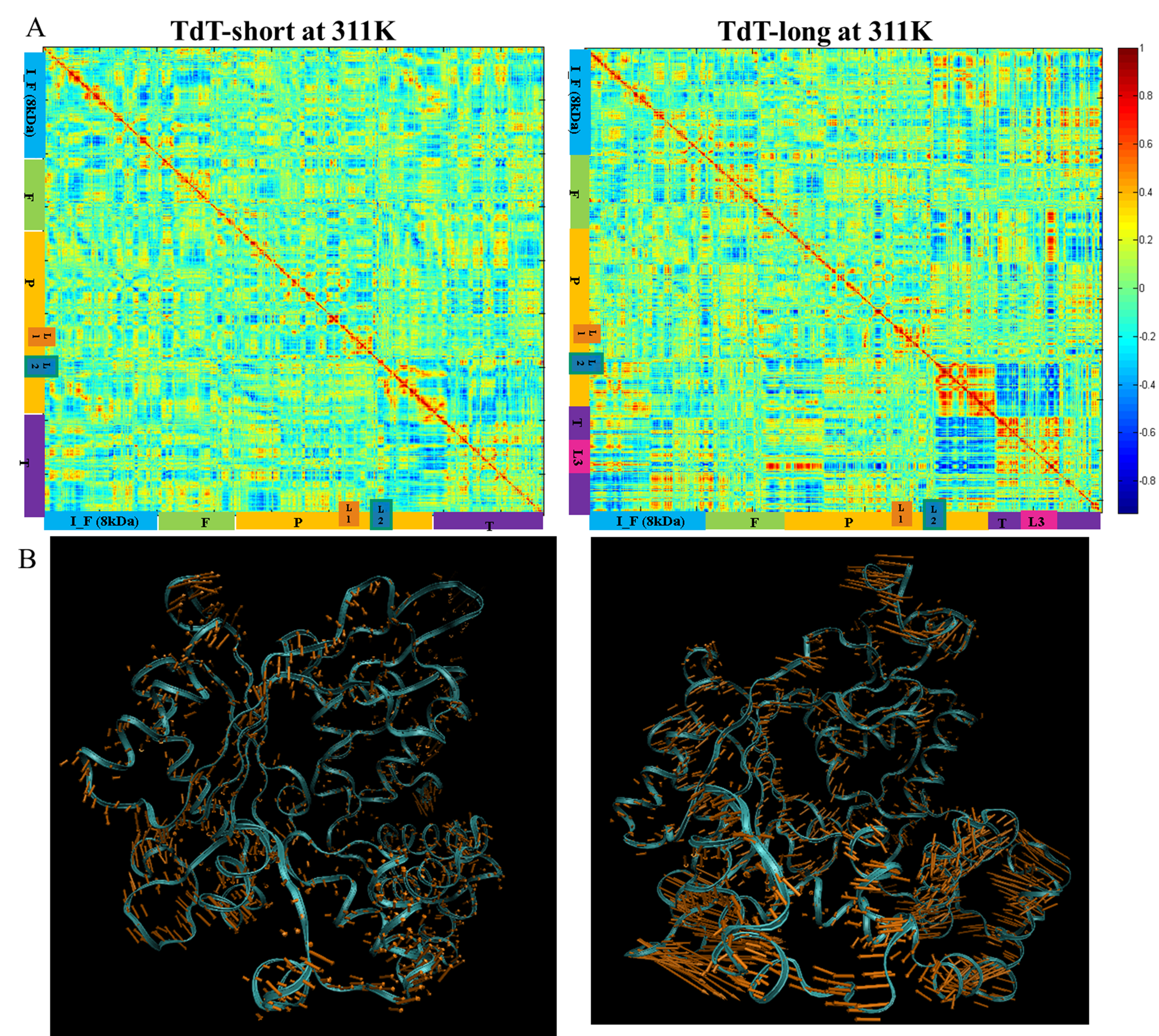

Supplement: S7 Fig — Increase in subdomain motions in TdT-long isoform as observed during simulations a) Comparison of correlation matrices of TdT-short and TdT-long at 311 K. Positive values (red spectrum) indicate correlated motion; negative values (blue spectrum) indicate anti-correlated motions between sub-domains. Higher correlations observed in TdT-long form than in TdT-short form. b) Arrow diagram of simulations at 311 K based on covariance matrices shows that TdT-short (left) does not undergo much fluctuations but TdT-long (right) has maximum motions in index finger, fingers and Loop3 regions (TIF) [file pone.0157286.s007.tif]

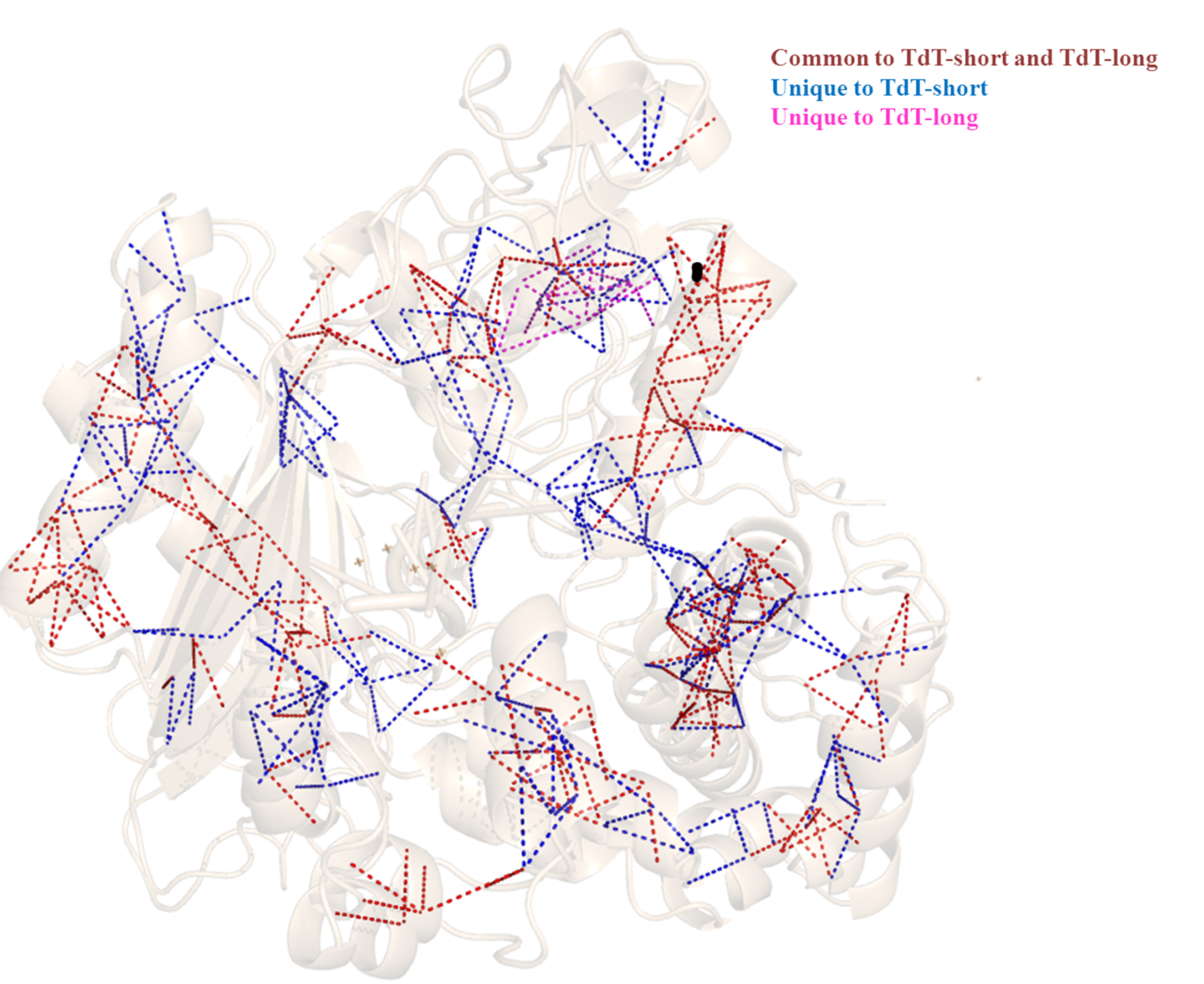

Supplement: S8 Fig — Common edges to form forms coloured in red, unique to only short form in blue and unique to only long form coloured in magenta. Most of the unique edges of cliques of TdT-long is located near thumb and Loop1 (TIF) [file pone.0157286.s008.tif]

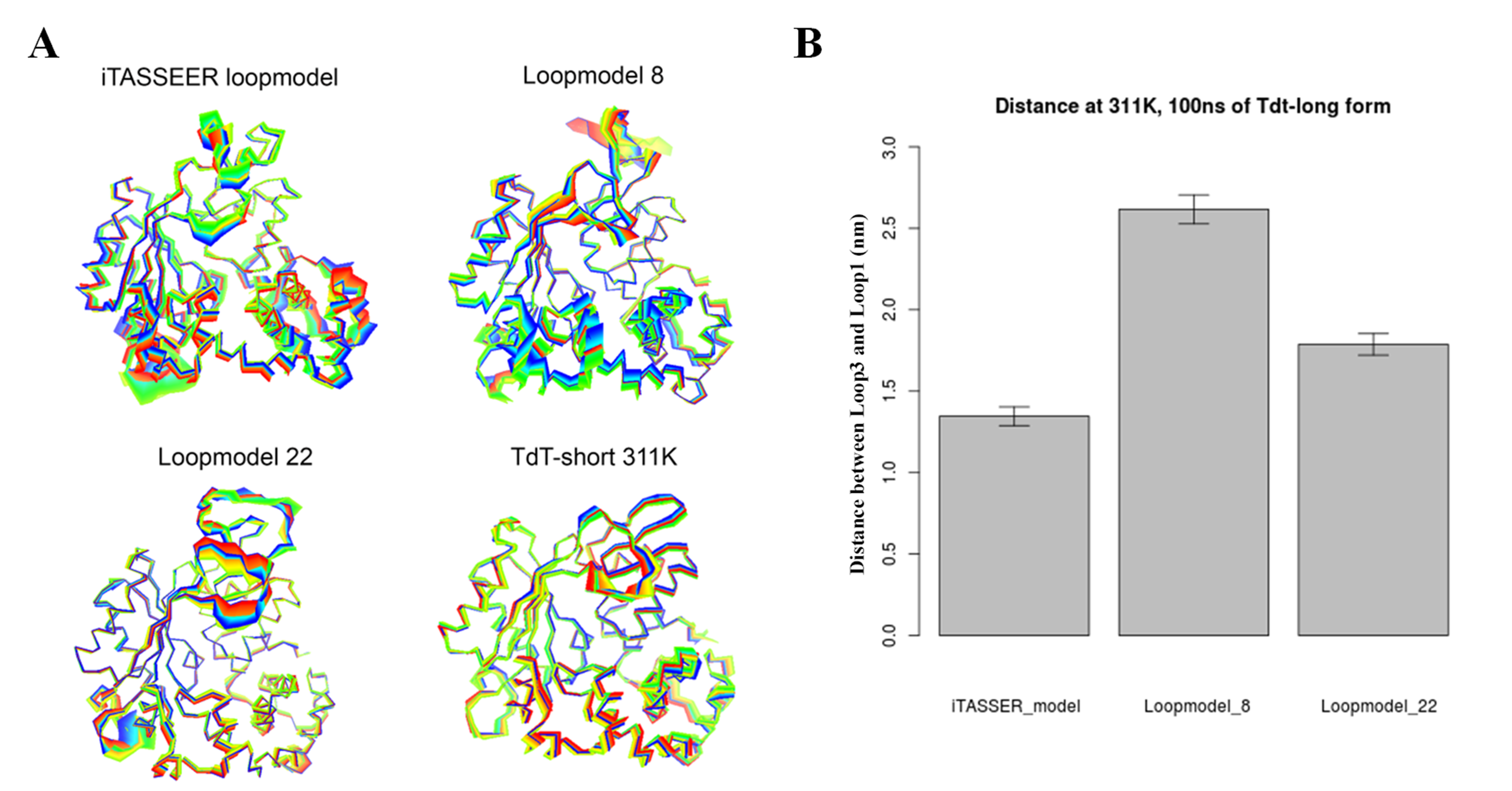

Supplement: S9 Fig — a) PCA from essential dynamics studies of simulations carried out at 311 K mapped onto the different loop conformers of TdT-long form (iTASSER loopmodel, Loopmodel8, Loopmodel 22) and TdT-short isoform (for comparison); b) Distances observed between of residues in Loop1 and Loop3 among various loopmodels of TdT-long form. (TIF) [file pone.0157286.s009.tif]

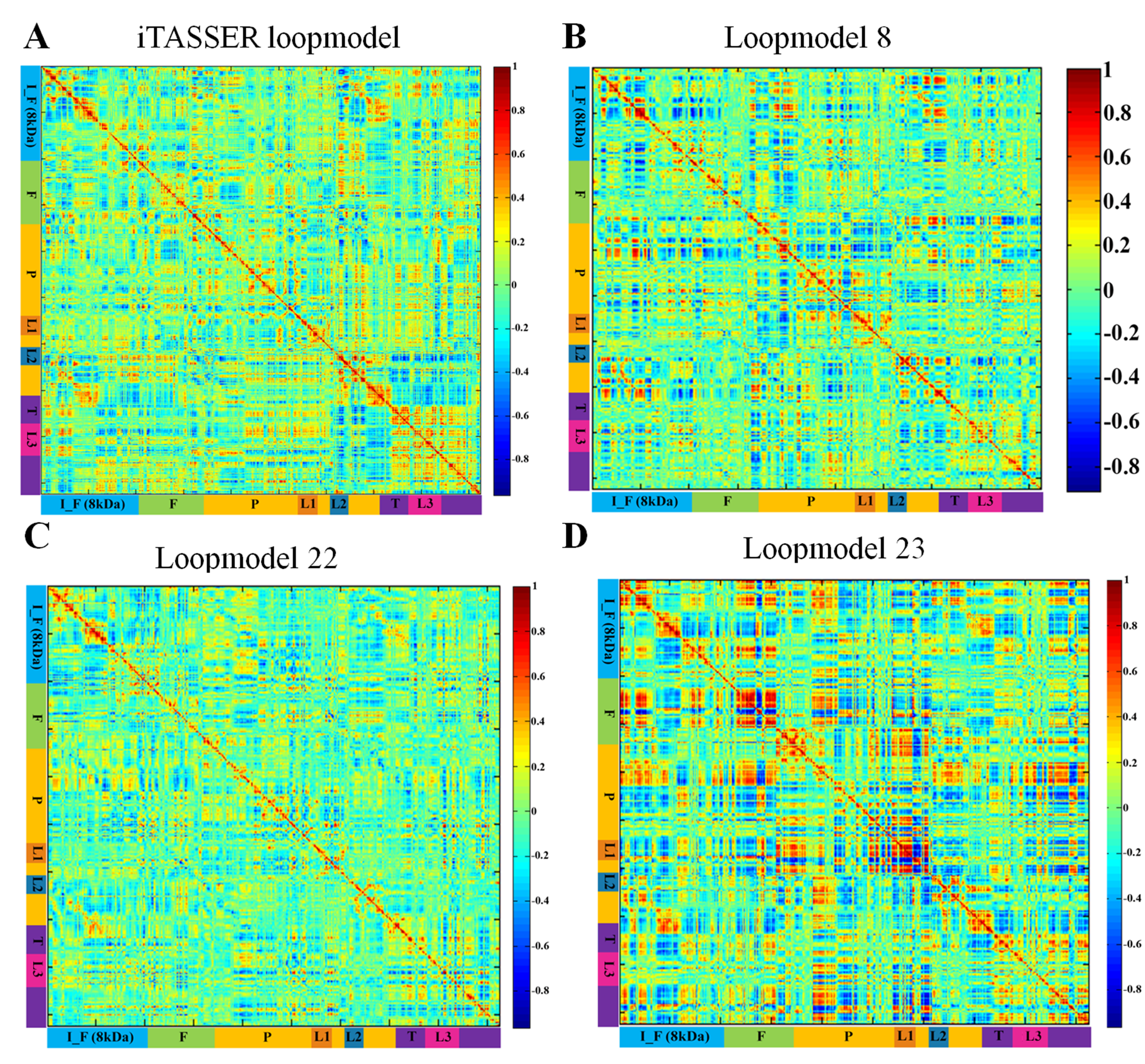

Supplement: S10 Fig — The top-ranking loop conformers of TdT-long isoform are a) iTASSER loopmodel, b) Loopmodel 8, c) Loopmodel 22 and d) Loopmodel 23 (from PRODAT library of SYBYL, Tripos package). Positive values (red spectrum) indicate correlated motion; negative values (blue spectrum) indicate anti-correlated motions between sub-domains. Sub-domains and loops of TdT has been marked on X-axis and Y-axis in different colours as: index finger or “I_F” (blue), fingers or “F” (green), palm or “P” (yellow), thumb or “T” (violet), Loop1 or “L1” (orange), Loop2 or “L2” (blue) and Loop3 or “L3” (magenta). Similar colour coded TdT-long structure has been provided at top left corner for ease in understanding. (TIF) [file pone.0157286.s010.tif]

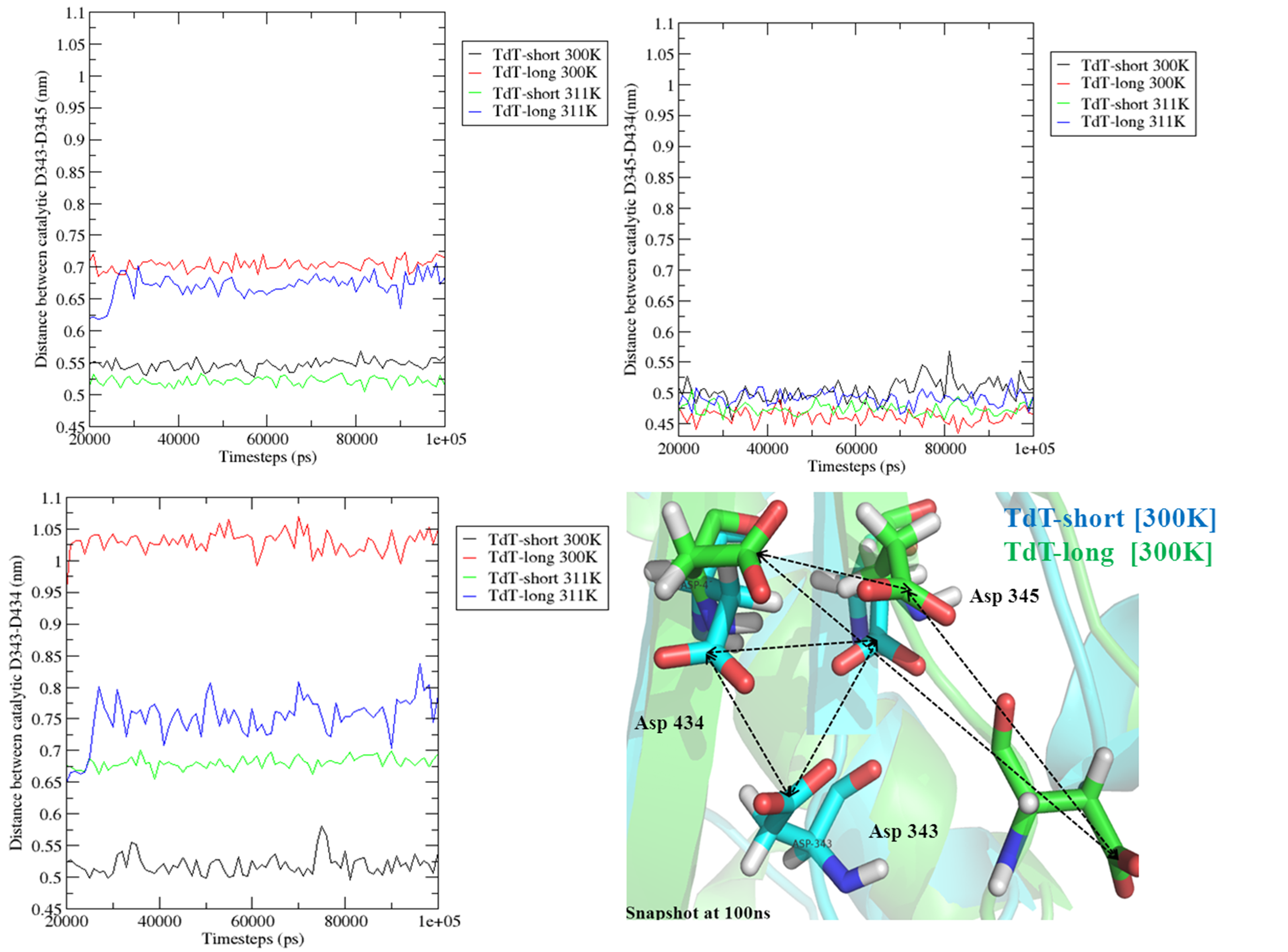

Supplement: S11 Fig — Snapshots of Asp343-Asp434 pair show longer distances within them in TdT-long isoform thus indicating distortion of active site triad. (TIF) [file pone.0157286.s011.tif]

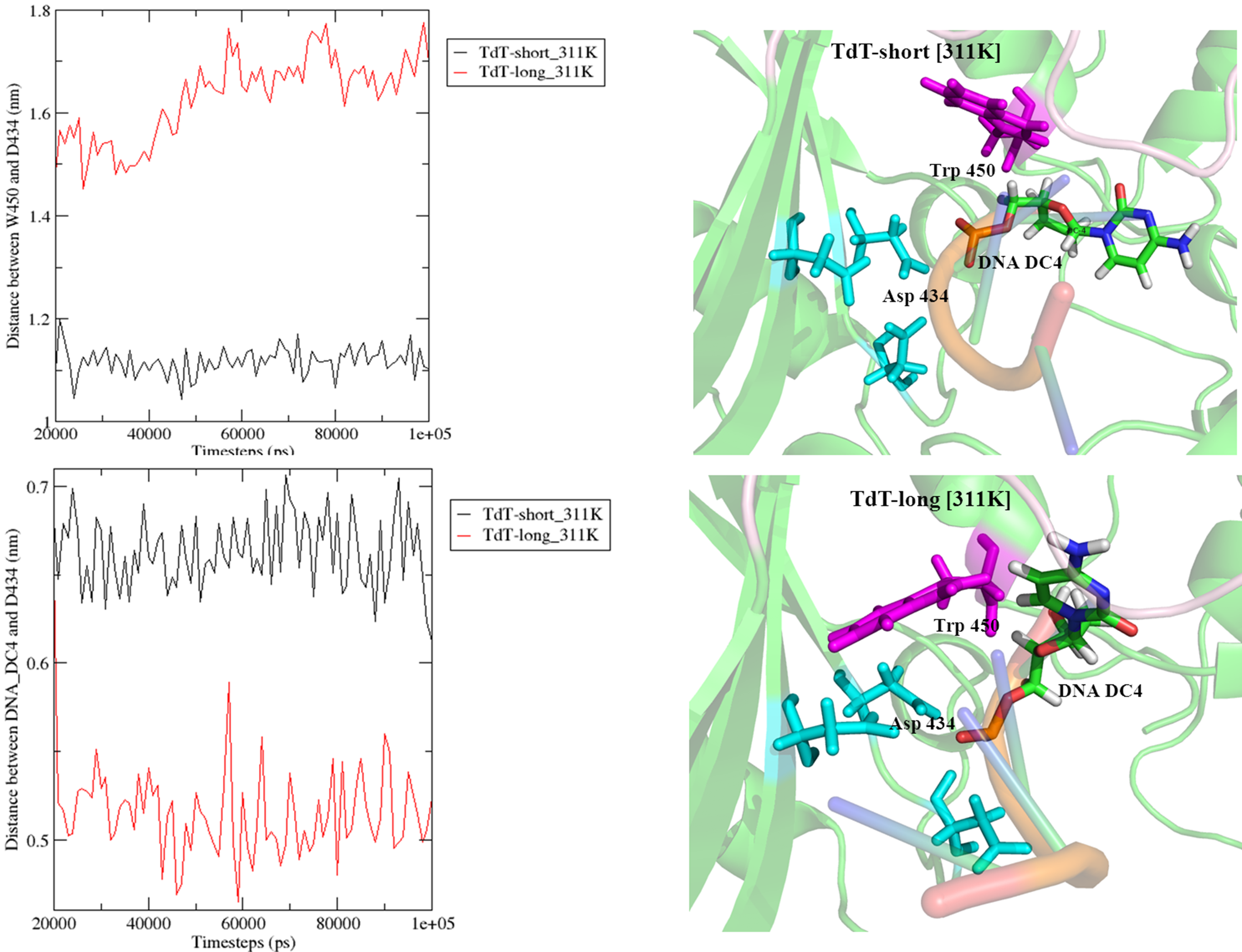

Supplement: S12 Fig — Distance of Trp450 from active site (D434) [upper left panel] and last nucleoside of DNA primer [lower left panel]. Orientation of Trp450 in short and long isoform of TdT [upper and lower right panel respectively]. (TIF) [file pone.0157286.s012.tif]

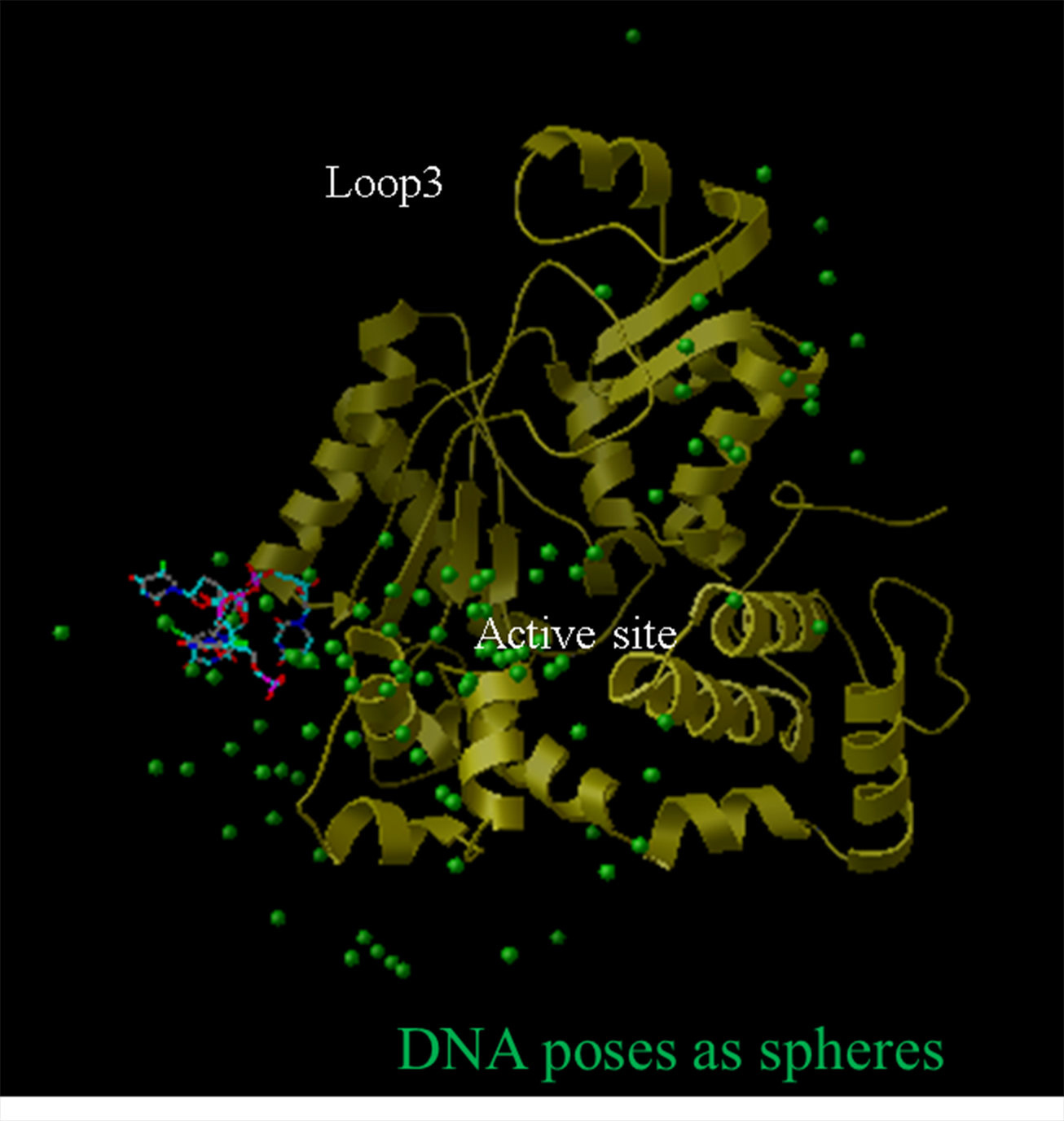

Supplement: S13 Fig — Lack of DNA primer docked to the Loop3 despite being high in positive charges depicts that the presence of insert did not alter the binding of DNA primer in the active site cavity. [Details referred in S1 File] (TIF) [file pone.0157286.s013.tif]

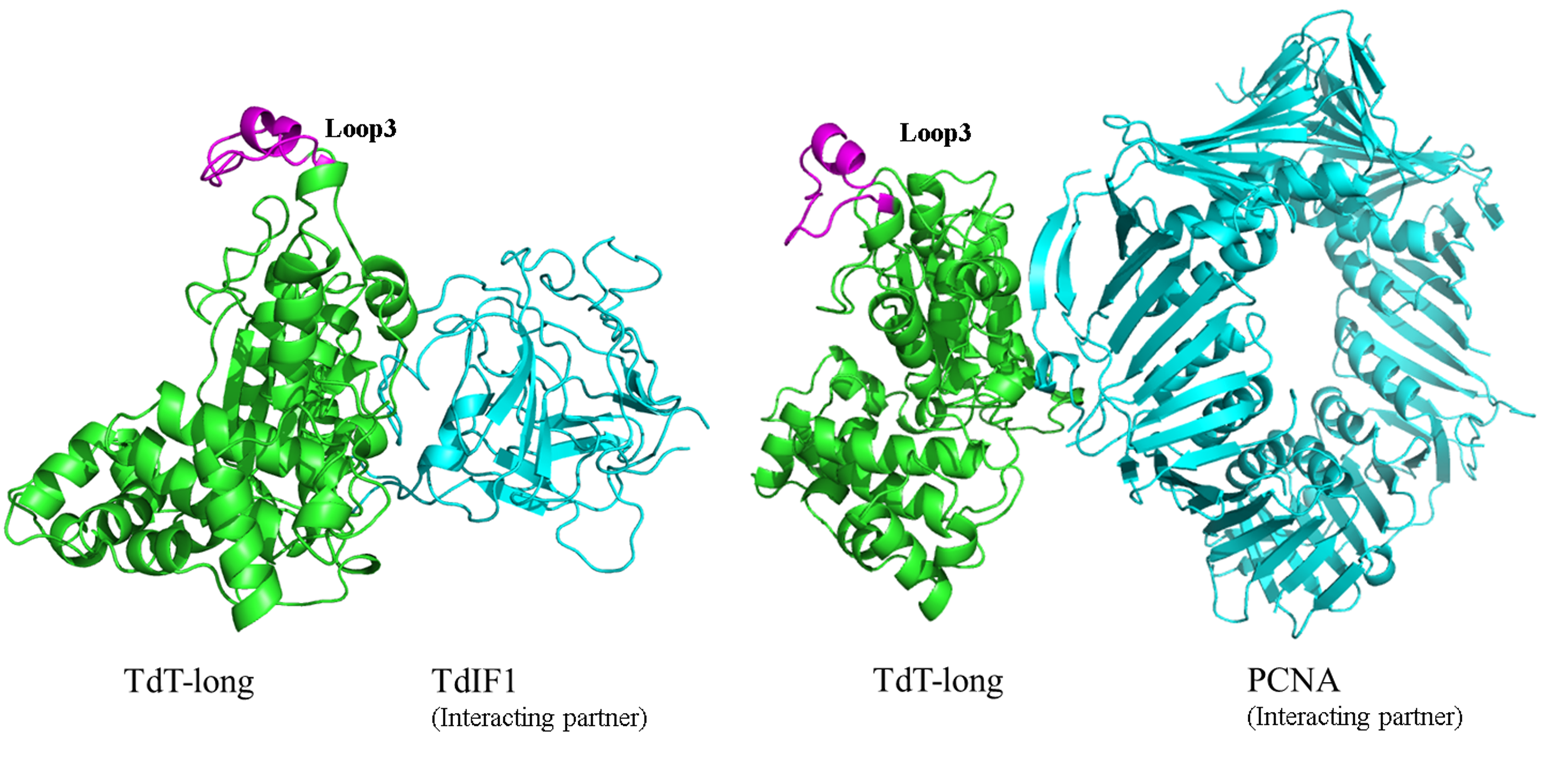

Supplement: S14 Fig — a) TdIF1 and b) PCNA near the thumb region. Binding at similar positions behind the thumb region support the theory that these proteins may competitively bind in order to regulate TdT. [Details referred in S1 File] (TIF) [file pone.0157286.s014.tif]

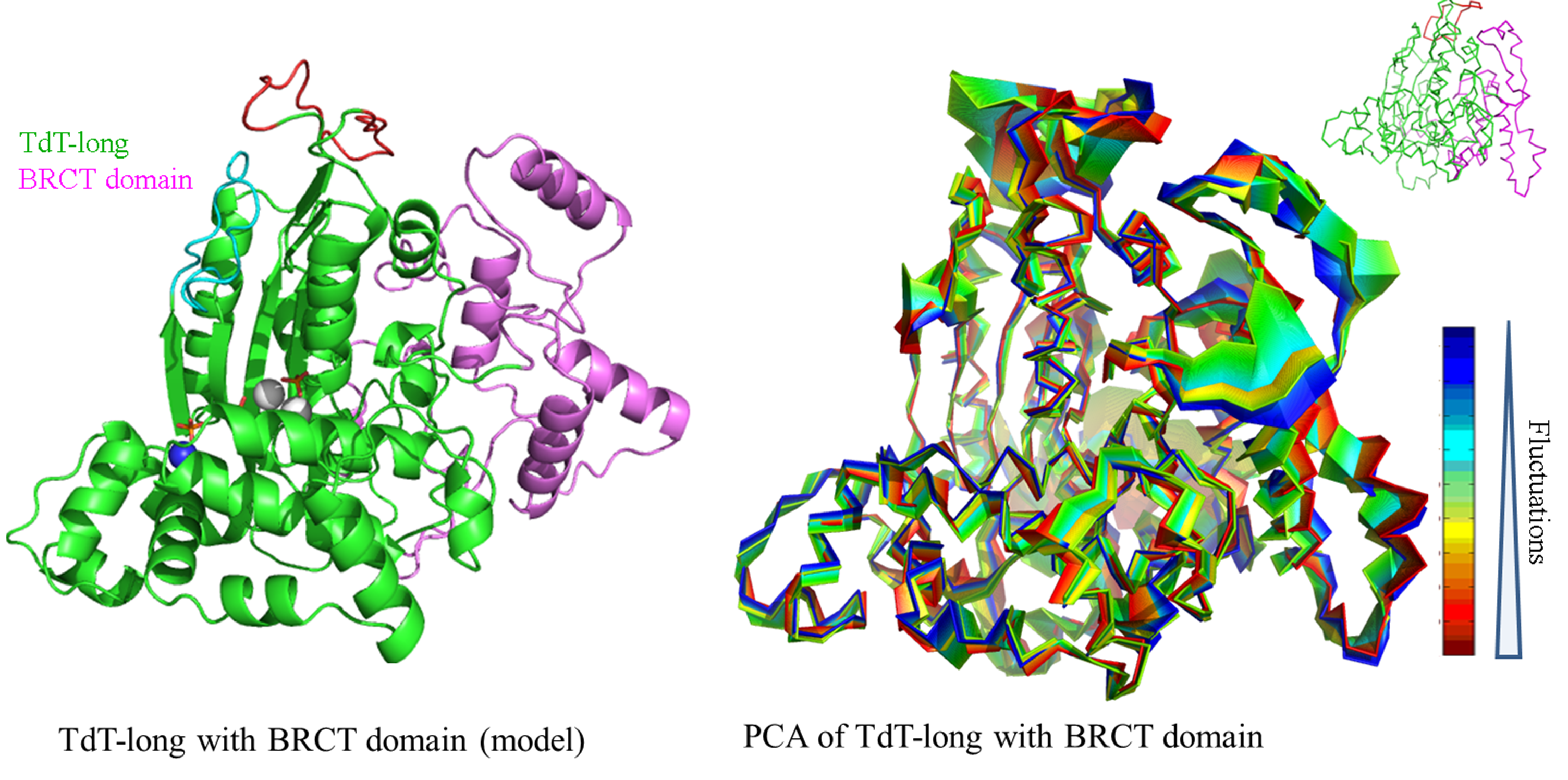

Supplement: S15 Fig — Effect of co-existing domain in TdT a) model of TdT-long with BRCT domain, b) PCA of simulation trajectories at 300 K mapped onto the model. [Details referred in S1 File] (TIF) [file pone.0157286.s015.tif]

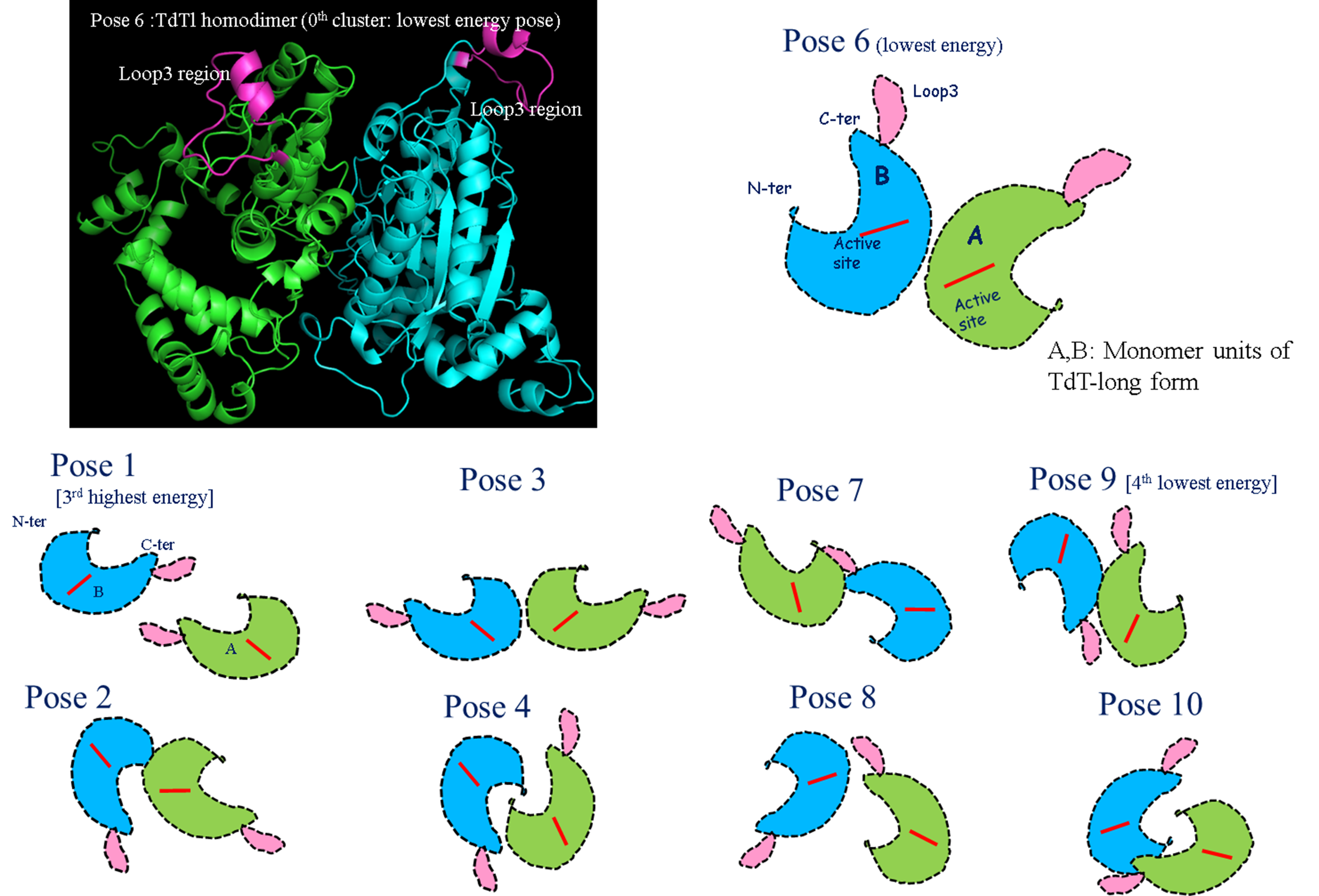

Supplement: S16 Fig — Loop3though having mostly hydrophobic residues and lying on surface is not found to lie in the dimer interface and hence does not make TdT-long form prone to homodimer formation. [Details referred in S1 File] (TIF) [file pone.0157286.s016.tif]

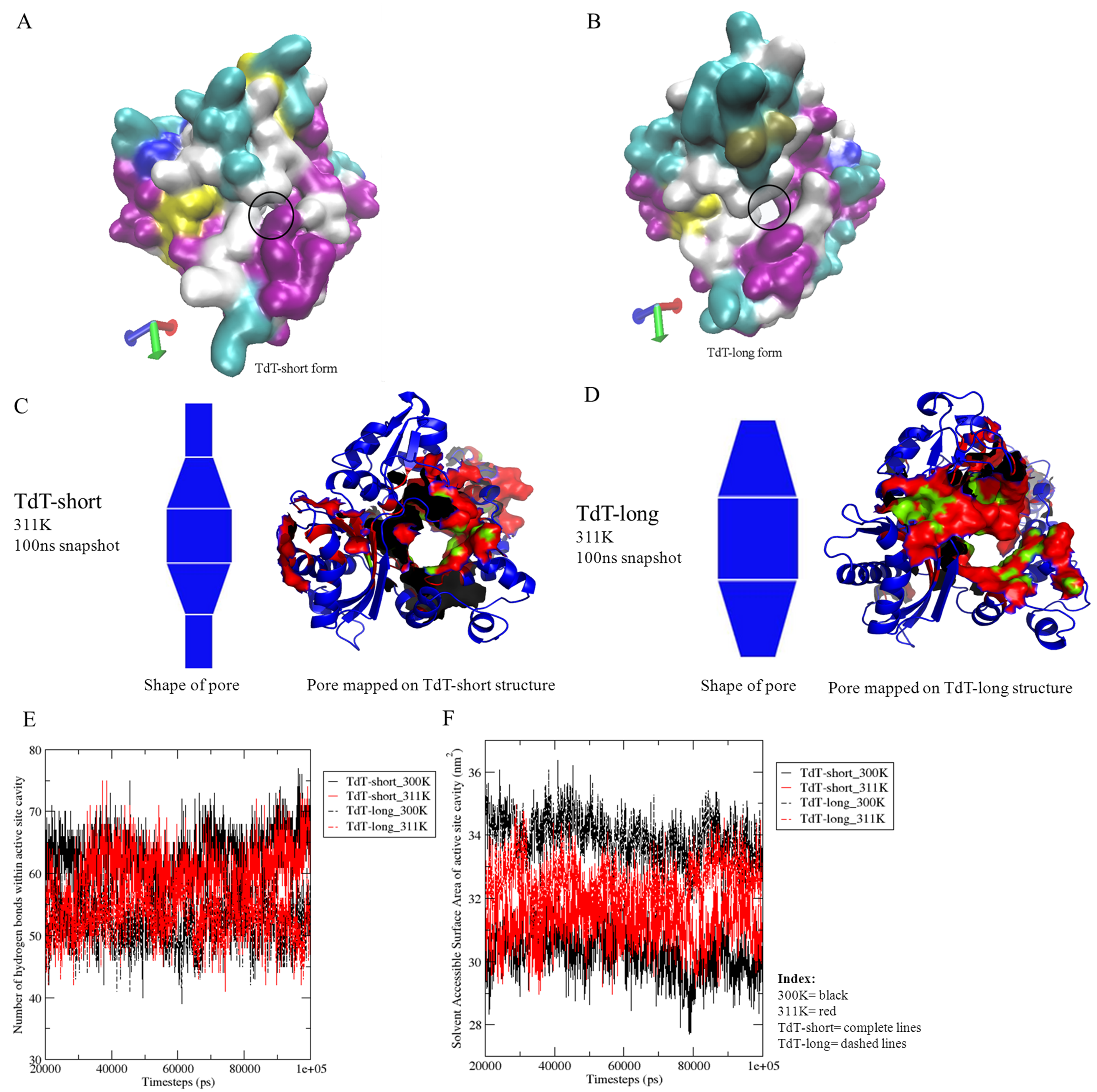

Supplement: S17 Fig — a, b)Surface representation of final snapshot of TdT-short and TdT-long form after simulations at 311 K temperature. Active site cavity/pore size of TdT-long form is wider than short form as circled in the figure. c)Pore shape and mapping of pore (red-green) onto TdT-short isoform and d) TdT-long isoform. Longer isoform has an expanded cavity/pore as compared to shorter isoform, e) Hydrogen bonds and f) solvent accessible surface area of the active site cavity for TdT-short (complete lines) and TdT-long isoforms (dashed lines) at physiological temperature of 300 K (black) and inactivation temperature of long form at 311 K(red). (TIF) [file pone.0157286.s017.tif]
