# Supplementary material for: Molecular Dynamics Simulations and Structural Analysis to Decipher Functional Impact of a Twenty Residue Insert in the Ternary Complex of Mus musculus TdT Isoform
Source: PLoS One. 2016 Jun 16;11(6):e0157286. doi: 10.1371/journal.pone.0157286 (PMC4911049; doi:10.1371/journal.pone.0157286)
Supplement: S1 File — Interaction of TdT with incoming DNA primer, BRCT domain and regulatory proteins and effect of presence of twenty residue insertion. (DOC) [file pone.0157286.s018.doc]

**SUPPLEMENTARY TEXT**

**Structural rationale and possible role of Loop3 in increasing thermosensitivity of TdT-long isoform with respect to TdT-short isoform**

**METHODS:**

**Network analysis:**

Various network properties *viz*. hubs, clusters and k-cliques were analyzed. ‘K-cliques’ is one such network component wherein if a network is created by “k” nodes, each node in such a clique is connected to each other by an edge. Edges represent the non-covalent interactions between two residues, while nodes represent amino acid residues. Thus detection of cliques conveys information about local subtle structural changes *viz*. formation of rigid areas in terms of global topology of protein structures.

**RESULTS:**

Conformational changes of Loop1 near the active site cavity

We analyzed the conformational changes occurring in Loop1 with respect to the active site in presence/absence of Loop3, by tracing distances between Loop1 (Asp396) and active site residue (Asp434). These were observed to be higher in TdT-short isoform, as compared to TdT-long isoform (Fig. 4e). Loop1 in the mutated TdT-long form, on the other hand, was farthest from the active site (unlike in TdT-long form) suggesting that the electrostatic interaction of Loop3 and Loop1 in wild-type TdT-long form positions Loop1 closer to the active site.

Conformational restraints of Loop1 region higher in TdT-long form

Various residues surrounding the Loop1 region are responsible for providing a rigid conformation to the loop in TdT short form [7,22,6,45]. However, these surrounding residues become flexible for making room for DNA template, in the case of template-dependent Pol μ. We have traced the movement of such residues with respect to nearest probable interacting residues from Loop1, throughout the simulations. Distance between Asp399-Lys403 pair (Asp399 belongs to Loop1 while Lys403 overlooked the active site) was comparatively less in TdT-long form(10.29±0.5Å compared to 11.71±0.48 Å). This supports the idea that Loop1 region may be more rigid (especially towards the active site) in TdT-long form than compared to TdT-short isoform (reduced RMSF (Fig. S2a) and shorter distance between the active site and Loop1 (Fig. 4e)).

Other functionally important residues (*viz*. Asp473 and His475), which may not be directly involved in the catalytic activity of TdT have been curated from the literature, mapped onto structure and followed during simulations [6,41,42]. They also showed increase in Loop1 restraints (Fig. S5)

Residues like Asp473 and His475 (from DNH motif of thumb subdomain) showed shorter distances (average values of 5.64±0.49 Å and 7.75±0.28 Å for Ser392-Asp473 and Phe401-His475 in TdT-long form while 6.40±0.70 Å and 8.32±0.45 Å was obtained for TdT-short form, respectively) with Loop1 residues in our simulations which may lead to higher rigidity of Loop1 in TdT-long isoform (Fig. S5).

Essential dynamics showed higher flexibility in the distant fingers and index-finger sub-domains

**PCA analyses:**

Top two dominant modes or principal components, when projected on the structures, showed that TdT-short form had least frequency of fluctuations in the core region (consisting of “palm” domain and part of “thumb” domain), while medium frequency motions were observed at Loop1, Loop2 and index finger region (Fig. 6). In TdT-long form, the dominant modes of PCA highlighted the presence of more fluctuations at fingers, index finger regions, apart from the Loop3-Loop1 pair (amplitude wise: covering larger space during motion) at high temperature (311K) [Extended simulations runs for 300ns depicted in Fig. S6]. Analyses of TdT-long form at high temperatures (i.e. inactivation temperature or higher) indicated that Loop3 and Loop1 fluctuated together "on a reduced frequency scale", while fingers and index finger regions fluctuated with very high amplitude and frequency. In case of TdT-short form, only Loop1 region showed significant movement with respect to other subdomains (Fig 6).

**Covariance matrices analyses:**

In case of TdT-long form, anti-correlated movements were observed between thumb regions and terminal portion of palm, while some part of the palm had highly correlated motion (movement in same direction) with the Loop3 embedded in the thumb. Correlated motion between Loop1 and Loop3 was also observed. (Fig. 5). The movements observed by thumb, index finger and fingers seem to be “claw-like”, which might be more pronounced due to the presence of Loop3. Thus, in case of TdT-long isoform, these results suggest that the presence of the distal Loop3 could provide higher flexibility to the fingers and index-finger regions of the TdT fold, thereby indirectly enhancing the thermosensitivity of the long isoform.

**Protein network analyses**

In order to trace the correlated movements present in TdT-long form, which may transcend from Loop3 to Loop1 to fingers to index finger, “protein residue networks” were further analyzed. Residue network analysis showed subdomains like thumb, index finger and fingers forming one major cluster (connected components of graph) in TdT-short isoform, but appeared as three different smaller clusters in TdT-long form (index finger and fingers formed separate cluster than thumb-palm region) (Fig. 7a). A similar trend was observed by creating residue networks on the basis of interaction strength, *Imin* [data not shown]). Increased density of cliques at various regions within a protein ascertains the strong connectivity in those areas. Unique cliques (k=3) were prevalent in dense clusters in TdT-short isoform, but cliques in TdT-long form were sparsely distributed. Moreover, unlike TdT-short form, lack of any cliques between thumb and index finger in TdT-long form, indicated that the latter did not possess a compact component of thumb and index finger. (Fig. 7b and Fig. S8). Distances measured between residues of thumb and index finger showed higher values in TdT-long form than TdT-short form, thus implying less-connected subdomains (Fig. 7c).

Another parameter related to protein function is the pI (isoelectric point), which for TdT-long isoform is 0.5 units higher than TdT-short isoform. This is due to the presence of five charged amino acid residues in Loop3, which may indirectly affect the temperature dependence of chemical equilibrium [52] and hence the thermosensitivity of TdT-long form.

Simulations of loop conformers of the modelled Loop3

Top-ranking loop conformers created for the TdT-long form (Please see Methods section) were also subjected to similar simulation and analysis studies. These models also show similar trends in behavior of Loop 1, Loop3 and the resultant fluctuations in the index-finger and finger subdomains as seen for the selected iTASSER model for TdT-long form. These trends have been illustrated via distance plots between Loop3 and Loop1, PCA and DCCM plots (Fig. S9, S10).

**Role of Loop3 in affecting polymerization rate and specific activity of TdT-long isoform**

Widening of active site cavity in TdT-long isoform

Active site region in TdT forms the largest cavity where the 4-mer DNA primer, two bivalent cations and incoming nucleotide along with the active site catalytic triad define the reaction center. Surface representation of final snapshots of TdT isoforms at 311K showed wider cavity/pore size (Fig. S17a,b). Investigations in confirming the shape of cavity showed that the cavity in the short isoform was optimally narrow in comparison to the long isoform (Supplementary Figure 17c,d). Higher solvent accessible surface area, volume and lower number of intra-protein hydrogen bonding could be observed within the active site cavity of TdT-long form as compared to TdT-short form, over different simulations which may imply a widening effect of this cavity (Fig. S17e,f).

**Interaction of TdT with incoming DNA primer, BRCT domain and regulatory proteins**

**Methods:**

**Docking DNA and with interacting partners:** The model of TdT-long form was docked to the DNA primer using semi-flexible blind docking option using AUTODOCK [S1]. Protein-protein docking was also performed on the model of TdT-long form with its interaction partners *viz.* TdIF1, PCNA by ClusPro docking method [S2].

**Modeling of BRCT domain in TdT-long model:** NMR structure of BRCT domain (PDB code: 2COE) [S3] was used as a template to model (using iTASSER) the BRCT domain with TdT-long form. The resultant model was further energy minimized using steepest descent method by SYBYL until convergence and simulated at 300K under similar conditions as described above.

**Results:**

With DNA primer: Docking studies of TdT-long isoform and DNA primer showed that the i20loop, despite being on the protein surface with a positive charged patch, did not alter the binding of the negatively charged DNA primer to its native binding site (i.e. at the active site cavity) (Fig. S13).

With interacting proteins: We consulted the STRING database [S4] and previous studies [S5-S7] for experimental evidence of TdT interacting with proliferating cell nuclear antigen (PCNA), TdT interacting factor 1 and 2 (TdIF1, TdIF2). It is known that while TdIF1 upregulated the activity of TdT, TdIF2 and PCNA played a role in its downregulation. These proteins were consecutively docked to TdT-long form, where i20loop was not observed in the interacting surface in any of the poses, which excluded the possibility of any interruptions in the protein-protein interactions of TdT-long form (Fig. S14). Our finding disagreed with the hypothesis made in earlier studies about interaction of i20loop with the interacting partners of TdT [S8].

With BRCT domain: TdT and its family members *viz.* polymerases λ and μ possess an extra BRCA1 C-terminal protein-protein interaction (BRCT) domain, which is demonstrated to be important for interaction with DNA and protein partners during Non-homologous end-joining (NHEJ) and VDJ recombination [45,47,48, S9]. This domain is also shown to interact with Ku heterodimer (S10). Due to its absence in the available TdT crystal structures, modeling and simulation studies of BRCT domain with TdT-long isoform was performed. The trajectories showed that while the loops in BRCT domain fluctuated heavily, there were no interactions with i20loop (Fig. S15).

Prospects for homodimerization: The i20loop is composed of mainly hydrophobic residues and five charged residues, out of which four Lysines lay on the surface of protein. Non-polar patches on the protein surface may lead to entropy-driven dimerization of protein domain [S11]. This idea that presence of hydrophobic patch on protein surface may lead to homodimerization of TdT-long form was tested by performing docking studies, where none of the top-ranking poses indicated i20loop to be present at the dimer interface and mediating the interaction between TdT monomers (Fig. S16).

DISCUSSION

Three-dimensional structure of TdT-short isoform from *Mus musculus* was solved by Delarue and co-workers in 2002. It was evident from the structure that TdT-short isoform adopts a closed conformation, unlike Pol β which undergoes an open-to-close subdomain conformational change after binding of incoming nucleotide.

Extended timescale runs of 300ns were also performed for TdT-long isoform at 311K to sample the conformational space better, but the results showed no major changes occurring between sampling at 100ns and 300ns. These results demonstrate that simulations of 100ns are sufficient for detailed analysis of TdT isoforms (Table 1). TdT-long isoform was prepared by modeling the twenty-residue Loop3 in the thumb subdomain of the shorter isoform [PDB: 1JMS] Validations of the starting model structure (iTASSER loopmodel) have been performed stringently and other top-ranking loop conformers (Loopmodels 8, 22, 23) have also been subjected to similar conditions in MD simulations in order to rule out the possibility of bias or error in Loop3 modeling.

In human Pol µ, loop1 has to be re-positioned to correctly bind to template DNA but in mouse TdT, Loop1 is more ordered and covers substantial part of the active site cavity (where DNA primer binding occurs) thus preventing DNA template binding (Fig. 1a).

The theories emerging from molecular dynamics calculations, presented here, are amenable to experimental verification *e.g.* elucidation of role of electrostatic interactions in binding of Loop 1 and Loop3 by performing site-directed mutagenesis studies. Further, theoretical methods like QM/MM, which combine quantum chemistry and molecular mechanics, can be utilized to quantify accurately the change in catalytic activity of TdT-long form [53].

REFERENCES

S1. Morris GM, Huey R, Lindstrom W, Sanner MF, Belew RK, Goodsell DS, et al. AutoDock4 and AutoDockTools4: Automated docking with selective receptor flexibility. J Comput Chem. 2009 Dec;30(16):2785–91.

S2. Comeau SR, Gatchell DW, Vajda S, Camacho CJ. ClusPro: an automated docking and discrimination method for the prediction of protein complexes. Bioinforma Oxf Engl. 2004 Jan 1;20(1):45–50.

S3. Nagashima, T, Hayashi, F., Yokoyama, S. Solution structure of BRCT domain of terminal deoxynucleotidyltransferase.RIKEN Structural Genomics/Proteomics Initiative (RSGI). RIKEN Structural Genomics/Proteomics Initiative (RSGI); 2005.

S4. Franceschini A, Szklarczyk D, Frankild S, Kuhn M, Simonovic M, Roth A, et al. STRING v9.1: protein-protein interaction networks, with increased coverage and integration. Nucleic Acids Res. 2013 Jan;41(Database issue):D808–15.

S5. Ibe S, Fujita K, Toyomoto T, Shimazaki N, Kaneko R, Tanabe A, et al. Terminal deoxynucleotidyltransferase is negatively regulated by direct interaction with proliferating cell nuclear antigen. Genes Cells. 2001;6(9):815–24.

S6. Fujita K, Shimazaki N, Ohta Y, Kubota T, Ibe S, Toji S, et al. Terminal deoxynucleotidyltransferase forms a ternary complex with a novel chromatin remodeling protein with 82 kDa and core histone. Genes Cells Devoted Mol Cell Mech. 2003 Jun;8(6):559–71.

S7. Yamashita N, Shimazaki N, Ibe S, Kaneko R, Tanabe A, Toyomoto T, et al. Terminal deoxynucleotidyltransferase directly interacts with a novel nuclear protein that is homologous to p65. Genes Cells Devoted Mol Cell Mech. 2001 Jul;6(7):641–52.

S8. Doyen N, Boulé J-B, Rougeon F, Papanicolaou C. Evidence that the long murine terminal deoxynucleotidyltransferase isoform plays no role in the control of V(D)J junctional diversity. J Immunol Baltim Md 1950. 2004 Jun 1;172(11):6764–7.

S9. Matsumoto T, Go K, Hyodo M, Koiwai K, Maezawa S, Hayano T, et al. BRCT domain of DNA polymerase μ has DNA-binding activity and promotes the DNA polymerization activity. Genes Cells Devoted Mol Cell Mech. 2012 Sep;17(9):790–806.

S10. Mahajan KN, Gangi-Peterson L, Sorscher DH, Wang J, Gathy KN, Mahajan NP, et al. Association of terminal deoxynucleotidyl transferase with Ku. Proc Natl Acad Sci. 1999 Nov 23;96(24):13926–31.

S11. Chong S-H, Ham S. Impact of chemical heterogeneity on protein self-assembly in water. Proc Natl Acad Sci U S A. 2012 May 15;109(20):7636–41.
